# Cyanobacterial Production of Biopharmaceutical and Biotherapeutic Proteins

**DOI:** 10.3389/fpls.2020.00237

**Published:** 2020-03-03

**Authors:** Nico Betterle, Diego Hidalgo Martinez, Anastasios Melis

**Affiliations:** Department of Plant and Microbial Biology, University of California, Berkeley, Berkeley, CA, United States

**Keywords:** fusion protein, interferon, therapeutic protein, transgene expression, *Synechocystis*

## Abstract

Efforts to express human therapeutic proteins in photosynthetic organisms have been described in the literature. Regarding microalgae, most of the research entailed a heterologous transformation of the chloroplast, but transformant cells failed to accumulate the desired recombinant proteins in high quantity. The present work provides methods and DNA construct formulations for over-expressing in photosynthetic cyanobacteria, at the protein level, human-origin bio-pharmaceutical and bio-therapeutic proteins. Proof-of-concept evidence is provided for the design and reduction to practice of “*fusion constructs as protein overexpression vectors*” for the generation of the bio-therapeutic protein interferon alpha-2 (IFN). IFN is a member of the Type I interferon cytokine family, well-known for its antiviral and anti-proliferative functions. Fusion construct formulations enabled accumulation of IFN up to 12% of total cellular protein in soluble form. In addition, the work reports on the isolation and purification of the fusion IFN protein and preliminary verification of its antiviral activity. Combining the expression and purification protocols developed here, it is possible to produce fairly large quantities of interferon in these photosynthetic microorganisms, generated from sunlight, CO_2_, and H_2_O.

## Introduction

Efforts to express human therapeutic proteins in photosynthetic microorganisms abound in the literature. In their preponderance, these entail heterologous transformation of microalgal chloroplasts as a synthetic biology platform for the production of biopharmaceutical and therapeutic proteins (Dyo and Purton, 2018, and references therein). The vast majority of such efforts have employed transformation of the chloroplast in the model green microalga *Chlamydomonas reinhardtii* via double homologous recombination of exogenous constructs encoding heterologous proteins (Demain and Vaishna, 2009; Surzycki et al., 2009; Tran et al., 2009; Coragliotti et al., 2011; Gregory et al., 2013; Jones and Mayfield, 2013; Rasala and Mayfield, 2015; Baier et al., 2018). A common feature of these efforts is the low yield of the transgenic proteins, rarely exceeding 1% of the total *Chlamydomonas reinhardtii* protein (Dyo and Purton, 2018). In general, there is a need to develop methods that will systematically and reliably over-express eukaryotic, including human therapeutic, proteins in photosynthetic microorganisms. The problem is exacerbated because of the frequent assumption in the field that a strong promoter will automatically cause gene overexpression when, in practice, SDS-PAGE fails to show presence of the transgenic protein and only sensitive Western blot analysis can offer evidence of low-levels of expression. A qualitative rule-of-thumb for overexpression in this respect is ability to detect the transgenic protein in SDS-PAGE analysis of total protein extracts.

Bacterial proteins can be heterologously over-expressed in cyanobacteria, reportedly up to 20% of total soluble protein, by using the strong *cpc* operon and possibly other endogenous or exogenous promoters (Kirst et al., 2014; Zhou et al., 2014; Formighieri and Melis, 2016; Vijay et al., 2019). Examples are afforded by Zhou et al. (2014), who described the function of a modified (partial) endogenous cyanobacterial promoter (*Pcpc560*), derived from the native cyanobacterial *cpc* operon promoter. They examined the efficacy of this promoter to express (i) the *trans*-enoyl-CoA reductase (Ter) protein from *Treponema denticola*, a Gram-negative and obligate anaerobic bacterium, and (ii) the D-lactate dehydrogenase (DldhE) protein from *Escherichia coli*. Both of these bacterial-origin genes and proteins were readily overexpressed in cyanobacteria under the control of the *Pcpc*. Kirst et al. (2014) showed that *Synechocystis* readily overexpressed, at the protein level and under the native *Pcpc*, the *nptI* gene from *E. coli*, encoding the neomycin phosphotransferase, a kanamycin resistance conferring protein. Similarly, Xiong et al. (2015) showed overexpression of the *Pseudomonas syringae efe* gene, encoding an ethylene forming enzyme, in *Synechocystis* sp. PCC 6803. Of interest, in this respect, is the demonstration of enhanced EFE protein accumulation upon transformation of *Synechocystis* with multiple copies of the *P. syringae efe* gene (Xiong et al., 2015). Likewise, Chaves and co-workers provided evidence that cyanobacteria will over-express, at the protein level, the *cmR* gene from *E. coli*, encoding a chloramphenicol resistance protein (Chaves et al., 2016), and the isopentenyl diphosphate isomerase (*fni*) gene from *Streptococcus pneumoniae*, either under the native *Pcpc* (Chaves et al., 2016) or heterologous *Ptrc* promoter (Chaves and Melis, 2018), strengthening the notion of relatively unhindered over-expression of heterologous bacterial genes in cyanobacteria. Evidence of over-expression in these cases was the visual detection and direct quantification of the transgenic proteins from the Coomassie-stained SDS-PAGE-resolved total cellular protein, offering a measure on the substantial presence of the recombinant protein.

However, recent experience has also shown that heterologous expression of eukaryotic plant and yeast genes occurs at low protein levels, regardless of the promoter used and mRNA levels achieved in the cyanobacterial cytosol (Formighieri and Melis, 2016). For example, plant terpene synthases could not be expressed well in cyanobacteria under the control of different strong endogenous and heterologous promoters (Formighieri and Melis, 2014; Englund et al., 2018). Heterologous expression in cyanobacteria of the isoprene synthase (Lindberg et al., 2010; Bentley and Melis, 2012), β-phellandrene synthase (Bentley et al., 2013), geranyl diphosphate (GPP) synthase from a higher plant origin (Bentley et al., 2014; Formighieri and Melis, 2017; Betterle and Melis, 2018), and the alcohol dehydrogenase (*ADH1*) gene from yeast (Chen and Melis, 2013), all showed low levels of recombinant protein expression, even under the control of strong endogenous (e.g., *psbA2, rbcL, cpc*) or strong heterologous promoters (e.g., *Ptrc*), and even after following a careful codon-use optimization of the target transgene (Lindberg et al., 2010; Bentley and Melis, 2012; Ungerer et al., 2012; Bentley et al., 2013; Chen and Melis, 2013; Formighieri and Melis, 2014; Englund et al., 2018). Similarly, only low levels of expression were reported for a chimeric complex of plant enzymes, including the ethylene synthase *efe* gene from *Solanum lycopersicum* (tomato) (Jindou et al., 2014; Xue and He, 2014), limonene synthase from *Mentha spicata* (spearmint) (Davies et al., 2014) and *Picea sitchensis* (Sitka spruce) (Halfmann et al., 2014b), the sesquiterpene farnesene and bisabolene synthases from *Picea abies* (Norway spruce) (Halfmann et al., 2014a) and *Abies grandis* (grand fir) (Davies et al., 2014). In these and other studies, transgenic protein levels were not evident on an SDS-PAGE Coomassie stain of protein extracts and, frequently, shown by sensitive Western blot analysis only, which was evidence for an admittedly low-level expression of plant-origin transgenes.

In separate work, Desplancq et al. (2005) showed that transgenic *Anabaena* sp. PCC 7120, a filamentous cyanobacterium, was able to express the *E. coli*, e.g., bacterial origin, maltose-binding protein (MBP), yielding up to 250 mg MBP per L culture. In further work, Desplancq et al. (2008) showed that *Anabaena* was also able to express 100 mg per L of gyrase B (GyrB), a 23 kD *E. coli* protein. This is consistent with the notion that cyanobacteria easily express other “bacterial” origin proteins. Animal-origin eukaryotic transgenes, however, are difficult to express in cyanobacteria. Desplancq et al. (2008) showed that the eukaryotic (human) oncogene E6 protein, when expressed in cyanobacteria, is toxic to the cells. Since efforts to express the oncogene E6 by itself failed due to toxicity of the product, Desplancq et al. (2008) undertook to express it as a fusion-protein with the highly-expressed maltose-binding protein as the leader sequence in an MBP^∗^E6 fusion. This effort resulted in a meager yield of 1 mg protein per L after 5 days of *nir* induction, i.e., 0.4% of the amount measured with MBP as the solo recombinant protein. They suggested that the MBP^∗^E6 fusion protein has an inhibitory effect on its own expression and further that this oncoprotein is toxic to *Anabaena* cells, evidenced from the about 50% inhibition in cell growth observed in the MBP^∗^E6 expressing transformants.

Interferons (IFNs) are a group of signaling proteins made and released by host cells in response to the presence of viruses. Interferons are named for their ability to “interfere” with viral infections of eukaryotic cells. Typically, a cell infected by a virus would release interferons causing adjacent cells to increase their anti-viral defenses. IFNs belong to the large class of proteins known as cytokines, used for communication between cells to trigger the protective defenses of the immune system that help eradicate pathogens (Parkin and Cohen, 2001). IFNs also activate immune cells, and increase host defenses by up-regulating antigen presentation. Interferon alpha-2 (IFN) is a member of the Type I interferon cytokine family, known for its antiviral and anti-proliferative functions. Recombinant *E. coli* (bacterial) expression of IFN resulted in the substantial formation of inclusion bodies, and required numerous purification and renaturing/refolding steps (Clark, 2001) that decreased the protein yield. Bis et al. (2014) described an expression and purification scheme for IFN using the pET-SUMO bacterial expression system and a single purification step. Using the SUMO protein, as the fusion tag, increased the soluble protein expression and minimized the amount of inclusion bodies in *E. coli*. Following protein expression, the SUMO tag was cleaved with the Ulp1 protease leaving no additional amino acids on the fusion terminus (Bis et al., 2014). The purified protein had antiviral and anti-proliferative activities comparable to the WHO International Standard, NIBSC 95/650, and the IFN standard available from PBL Assay Science.

There is a need to develop recombinant DNA technologies for the generation of low-cost biopharmaceutical proteins, without relying on animal systems, and without causing depletion of natural resources, emission of greenhouse gases, or other environmental degradation. In this respect, a direct photosynthetic production of such compounds is promising. Recent work from this lab contributed with the design of oligonucleotide fusion constructs as protein overexpression vectors that could be used in cyanobacteria for the over-expression of recalcitrant plant, animal, and human genes. It was successfully applied in the over-expression of transgenic terpene synthases from a variety of plants in these photosynthetic microorganisms. The barrier to expressing eukaryotic plant proteins in cyanobacteria at high levels was thus overcome by the fusion-constructs technology (Formighieri and Melis, 2015, 2016; Chaves et al., 2017; Betterle and Melis, 2018, 2019). In this approach, highly-expressed endogenous cyanobacteria genes, such as the *cpcB* gene, encoding the β-subunit of phycocyanin, or highly-expressed heterologous genes, such as the *nptI* gene, encoding the kanamycin resistance protein, have successfully served as leader sequences in the fusion formulation, resulting in the accumulation of eukaryotic proteins up to ∼20% of the total cyanobacterial protein (Formighieri and Melis, 2015, 2016). This fusion construct technology was successfully applied in this work to enable accumulation in *Synechocystis* of the human interferon, serving as a proof of principle in the cyanobacterial synthesis and accumulation of biopharmaceutical proteins.

## Materials and Methods

### *Synechocystis* Strains, Recombinant Constructs, and Culture Conditions

The cyanobacterium *Synechocystis* sp. PCC 6803 (*Synechocystis*) was used as the experimental strain in this work and referred to as the wild type (WT). A gene sequence encoding the human interferon α-2 protein (hereafter referred to as IFN)^1^, without the corresponding N-terminal signal peptide, was codon optimized for protein expression in *Synechocystis* using an open software system^2^. DNA constructs for *Synechocystis* transformation were synthesized by Biomatik USA (Wilmington, DE, United States). Sequences of the DNA constructs are shown in the Supplementary Material.

*Synechocystis* transformations were carried out according to established protocols (Williams, 1988; Lindberg et al., 2010; Eaton-Rye, 2011). Wild type and transformants were maintained on BG11 media supplemented with 1% agar, 10 mM TES-NaOH (pH 8.2) and 0.3% sodium thiosulfate. Liquid cultures of BG11 were buffered with 25 mM sodium bicarbonate, pH 8.2, and 25 mM dipotassium hydrogen phosphate, pH 9, and incubated in the light upon slow continuous bubbling with air at 26°C. Transgenic DNA copy homoplasmy in the cells was achieved upon transformant incubation on agar in the presence of increasing concentrations of chloramphenicol (3–25 μg/mL). Growth of the cells was promoted by using a balanced combination of white LED bulbs supplemented with incandescent light to yield a final Photosynthetically Active Radiation (PAR) intensity of ∼100 μmol photons m^–2^ s^–1^.

### Genomic DNA PCR Analysis of *Synechocystis* Transformants

**FIGURE 1:**
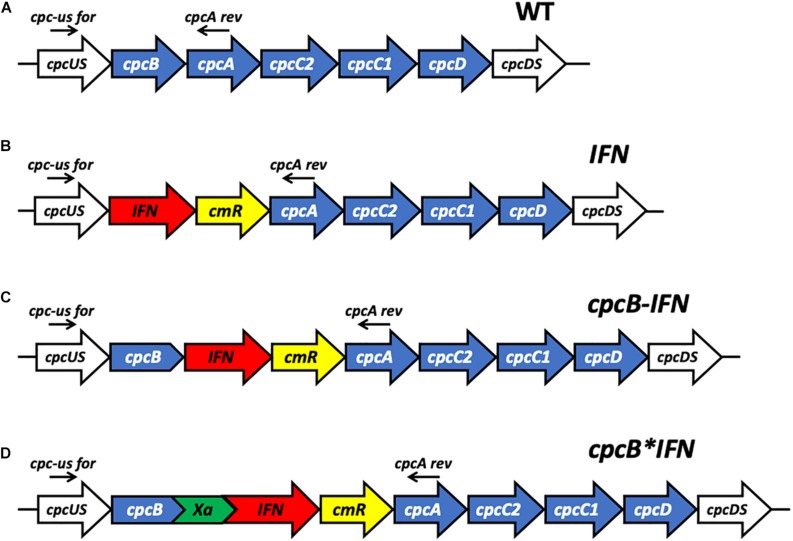
Schematic overview of DNA constructs designed for the transformation of the *Synechocystis* PCC 6803 (*Synechocystis*) genome. **(A)** The native *cpc* operon, as it occurs in wild type S*ynechocystis*. This DNA sequence and associated strain are referred to as the wild type (WT). **(B)** Insertion in the *cpc* operon of the codon-optimized human interferon (*IFN*) gene followed by the chloramphenicol (*cmR)* resistance cassette in an operon configuration, replacing the phycocyanin-encoding β-subunit *cpcB* gene of *Synechocystis*. This DNA construct is referred to as *IFN*. **(C)** Insertion in the *cpc* operon of the codon-optimized *IFN* gene immediately downstream of the phycocyanin-encoding β-subunit *cpcB* gene of *Synechocystis*, followed by the *cmR* resistance cassette, in an operon configuration. This DNA construct is referred to as *cpcB-IFN*. **(D)** Insertion in the *cpc* operon of the codon-optimized *IFN* gene as a fusion construct with the phycocyanin-encoding β-subunit *cpcB* gene, with the latter in the leader sequence position. The fusion construct *cpcB^∗^IFN* was followed by the *cmR* resistance cassette in an operon configuration. *cpcB* and *IFN* genes were linked by the DNA sequence encoding the Factor Xa cleavage site. The latter comprises the Ile-Glu/Asp-Gly-Arg amino acid sequence. This DNA construct is referred to as the *cpcB^∗^IFN*.

Genomic DNA templates were prepared, as previously described (Formighieri and Melis, 2014). A 20 μL culture aliquot was provided with an equal volume of 100% ethanol followed by brief vortexing. A 200 μL aliquot of a 10% (w/v) Chelex^®^100 Resin (BioRad) suspension in water was added to the sample prior to mixing and heating at 98°C for 10 min to lyse the cells. Following centrifugation at 16,000 *g* for 10 min to pellet cell debris, 5 μL of the supernatant was used as a genomic DNA template in a 25 μL PCR reaction mixture. Q5^®^ DNA polymerase (New England Biolabs) was used to perform the genomic DNA PCR analyses. A list of primers used is given in the Supplementary Table S1. Transgenic DNA copy homoplasmy in *Synechocystis* was tested using suitable primers listed in the Supplementary Material. The genomic DNA location of these primers is indicated in Figure 1 for the appropriate DNA constructs.

### Protein Analysis

Cells in the mid exponential growth phase (OD_730_ ∼1) were harvested by centrifugation at 4,000 *g* for 10 min. The pellet was resuspended in a solution buffered with 25 mM Tris-HCl, pH 8.2, also containing a cOmplete^TM^ mini protease inhibitor cocktail (Roche; one 50 mg tablet was added per 50 mL suspension). Cells were broken by passing the suspension through a French press cell at 1,500 psi. A slow speed centrifugation (350 *g* for 3 min) was applied to remove unbroken cells. For protein electrophoretic analysis, sample extracts were solubilized upon incubation for 1 h at room temperature in the presence of 125 mM Tris-HCl, pH 6.8, 3.5% SDS, 10% glycerol, 2M urea, and 5% β-mercaptoethanol. SDS-PAGE was performed using Mini-PROTEAN TGX precast gels (BIORAD). Densitometric quantification of target proteins, as a percentage of the total cellular protein, was performed using the BIORAD (Hercules, CA, United States) Image Lab software. A subsequent Western blot analysis entailed transfer of the SDS-resolved proteins to a 0.2 μm pore size PVDF membrane (Life Technologies, Carlsbad, CA, United States). Protein transfer to PVDF was followed by protein probing with rabbit-raised CpcA specific polyclonal antibodies (Abbiotec, San Diego, CA, United States), as previously described (Formighieri and Melis, 2015), or IFN-specific polyclonal antibodies (Abcam, Cambridge, MA, United States).

### Recombinant Protein Purification

Total cellular extracts (concentration 100 μg dcw mL^–1^) from wild-type and transformant strains of *Synechocystis* were gently solubilized upon incubation with 1% Triton X-100 at 0°C for 20 min. Solubilization of the extracts was conducted in an ice-water bath, upon gentle shaking. Following this solubilization treatment, samples were centrifuged at 10,000 *g* for 10 min to remove cell debris and insoluble material. His-Select resin (Sigma, St. Louis, MO, United States) was employed as a solid phase for protein binding and purification through cobalt affinity chromatography. Manufacturer’s instructions were followed for both batch-type and column-based binding and purification. The washing solution was buffered with 20 mM Hepes, pH 7.5, and contained 150 mM NaCl and 10 mM imidazole to help remove non-target proteins. The elution solution was buffered with 20 mM Hepes, pH 7.5, and contained 150 mM NaCl and 250 mM imidazole to elute target protein from the resin.

### Zn-Staining

SDS-PAGE was incubated in 5 mM zinc sulfate for 30 min (Li et al., 2016). To detect covalent chromophore-binding polypeptides, zinc induced fluorescence was monitored by Chemidoc imaging system (BIORAD), employing UV light as a light source. Loading of total protein extracts was the same as for the Coomassie-stained SDS-PAGE.

### Interferon Activity

Viruses replicate by co-opting normal host cell functions, turning cells into viral factories. Interferon protects cells by binding to extracellular receptors activating a cascade of signals that shuts down both *de novo* protein and DNA synthesis, depriving the invader the means to replicate. This puts the cells into a semi dormant state, preventing the production of new virus. This is most evident in the life cycle of lytic viruses, which normally burst or lyse target cells, but fail to do so when cells are in an interferon-induced antiviral state. Accordingly, one can assess interferon activity by visually comparing the number of intact/lysed cells for a particular concentration of interferon added (Rubinstein et al., 1981; Budd et al., 1984; Crisafulli et al., 2008).

A cytopathic effect (CPE) protection assay was employed to assess the capacity of cyanobacteria-derived interferon to lower viral infectivity. Human U20S osteosarcoma cells were seeded onto 24 well plates at a concentration of 5 × 10^5^ cells per well and incubated overnight at 37°C and 5% CO_2_. When confluent, cells were incubated with serial dilutions ranging from 1 × 10^–3^ to 1 × 10^–7^ μg/mL of either commercial interferon (IFN alpha 2 Cat. #11100-1 PBL Assay Science, Piscataway, NJ, United States), cyanobacteria-derived recombinant interferon Cpcb^∗^His^∗^IFN, or control diluent for 24 h prior to addition of vesicular stomatitis virus (VSV) (1 × 10^7^) (pfu/mL), diluted to a final concentration of 200 plaques per well and incubated for 120 min at 37°C. Plaques were stabilized by adding premixed 2% methyl cellulose in 2x Dulbecco’s modified Eagle’s medium (DMEM) overlaid onto each well and incubated at 37°C overnight. At 24 h post-infection incubation with VSV, media were removed, cell monolayers were rinsed in PBS and stained using crystal violet (4% formaldehyde, glycerol and 0.5% crystal violet) for 1 h. Crystal violet stain was then removed, stained plates were washed in water and plaques subsequently counted.

## Results

### *cpcB^∗^IFN* Fusion Constructs

Case study of this experimental work is the heterologous expression of the mature human interferon α-2 protein^3^, hereafter referred to as IFN, in the model cyanobacteria *Synechocystis* sp. PCC 6803 (*Synechocystis*). To validate the fusion constructs approach, three different DNA constructs were designed for the transformation of wild type (WT) *Synechocystis* through double homologous DNA recombination in the *cpc* operon locus (Figure 1A). Construct *IFN* (Figure 1B) was codon optimized for expression in *Synechocystis*, and designed to replace the *cpcB* gene in the *cpc* operon. In this case, *IFN* was followed by the chloramphenicol resistance cassette (*cmR*) in an operon configuration. Construct *cpcB-IFN* (Figure 1C) was designed to insert both the *IFN* and the *cmR* genes after the c*pcB* gene in an operon configuration. Finally, construct *cpcB^∗^IFN* (Figure 1D) was conceived to replace the *cpcB* gene in the *cpc* operon with the fusion construct *cpcB*^∗^*IFN*, followed by the *cmR* gene in an operon configuration. It is noteworthy that the Factor Xa cleavage-encoding sequence was inserted between the *cpcB* and *IFN* genes in the construct of Figure 1D. Factor Xa cleavage site was chosen because Factor Xa protease activity would free the N-terminus of the target protein, thus allowing recovery of the natural product (Kavoosi et al., 2007). The precise nucleotide sequence and spacers of these constructs is given in the Supplementary Material of this work. The above-described genetic manipulations caused a disruption in the physiological expression of the *cpc* operon and prevented the assembly of the phycocyanin peripheral antenna in the cyanobacteria. It is known that the *cpcB* gene and, in fact, the entire *cpc* operon can be deleted from the cyanobacterial genome, resulting in a smaller phycobilisome light-harvesting antenna size and requiring a higher light intensity for the saturation of photosynthesis but entailing no adverse cell or photosynthesis fitness effects (Ajlani and Vernotte, 1998; Page et al., 2012; Liberton et al., 2013; Kirst et al., 2014; Formighieri and Melis, 2015; Chaves et al., 2016).

**FIGURE 2:**
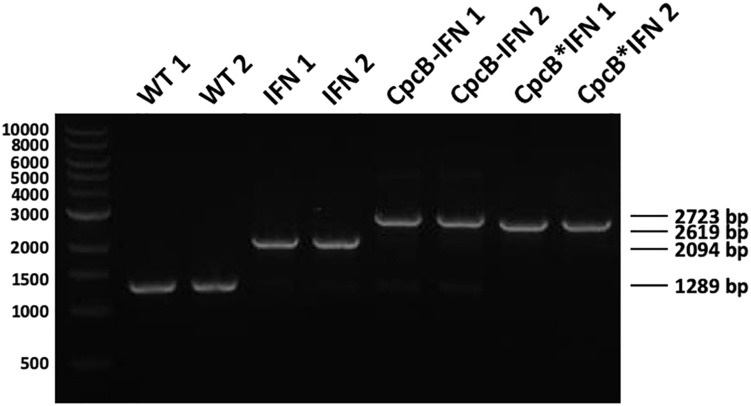
Genomic DNA PCR analysis testing for transgenic DNA copy homoplasmy in *Synechocystis* transformants. Wild type and transformant strains were probed in genomic DNA PCR reactions for product generation and transgenic DNA segregation. Primers <*cpc-us for*> and <*cpcA rev*> showed substantially different and unique products in the wild type and the different transformants comprising the constructs of Figure 1. Wild type PCR products had a 1,289 bp size, whereas the IFN, cpcB-IFN, and the cpcB^∗^IFN transformants generated 2,094, 2,723, and 2,619 bp size products, respectively. Absence of wild type products from the latter was evidence of DNA copy homoplasmy for the transformants. (The cpcB-IFN construct generated a product size slightly larger than that of the cpcB^∗^IFN because it contained the *Synechocystis* native cpcB-cpcA intergenic DNA sequence. Please see gene nucleotide sequences in the Supplementary Material).

Attainment of transgenic DNA copy homoplasmy in the three transformant strains was tested through genomic DNA PCR analysis. Primers *cpc-us for* and *cpcA rev* were designed on the flanking regions of the transgenic DNA insertion sites (Figure 1). PCR amplification using WT genomic DNA as a template generated a product of 1,289 bp (Figure 2). PCR amplification using DNA from the transformant IFN, CpcB-IFN, and CpcB^∗^IFN strains generated the expected product sizes of 2,094, 2,723, and 2,619 bp, respectively. Attainment of DNA copy homoplasmy was evidenced by the absence of WT PCR products in the PCR amplification reactions of the IFN transformants.

**FIGURE 3:**
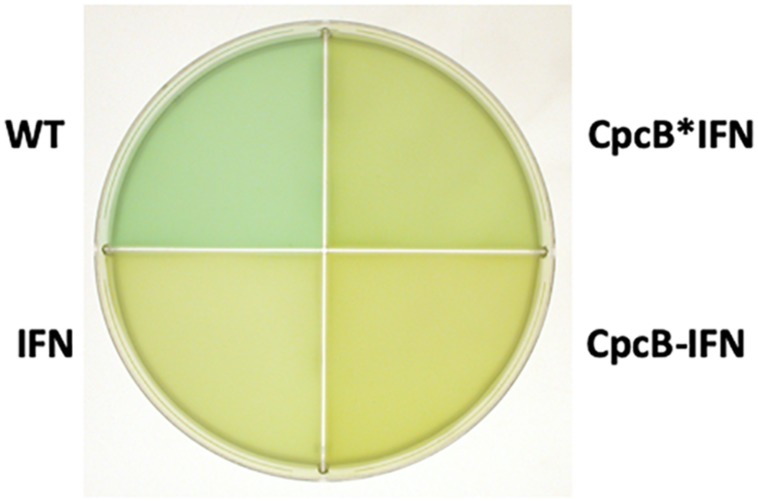
Coloration of cells from photoautotrophically-grown liquid cultures showing a blue–green wild type (WT) phenotype, and greenish phenotype for the IFN, CpcB-IFN, and CpcB^∗^IFN-containing transformants. The latter did not assemble phycocyanin rods, hence the absence of the distinct blue cyanobacterial coloration from the cells.

Upon attainment of transgenic DNA copy homoplasmy, WT and transformant strains were grown photo-autotrophically in liquid BG-11 cultures. The visual phenotype was noticeably different between the WT and transformant strains, as shown in Figure 3. The WT cells had a blue–green coloration, consistent with the presence of blue phycocyanin and green chlorophyll pigments in their functional light-harvesting antennae. All transformant strains showed a yellow-green pigmentation, suggesting lack of phycocyanin, which is responsible for the blue pigmentation of the cells. This is consistent with previous results in the literature (Ajlani and Vernotte, 1998; Page et al., 2012; Kwon et al., 2013; Liberton et al., 2013; Kirst et al., 2014; Formighieri and Melis, 2015; Chaves and Melis, 2018) and underscores the absence of assembled phycocyanin rods in the transformants.

**FIGURE 4:**
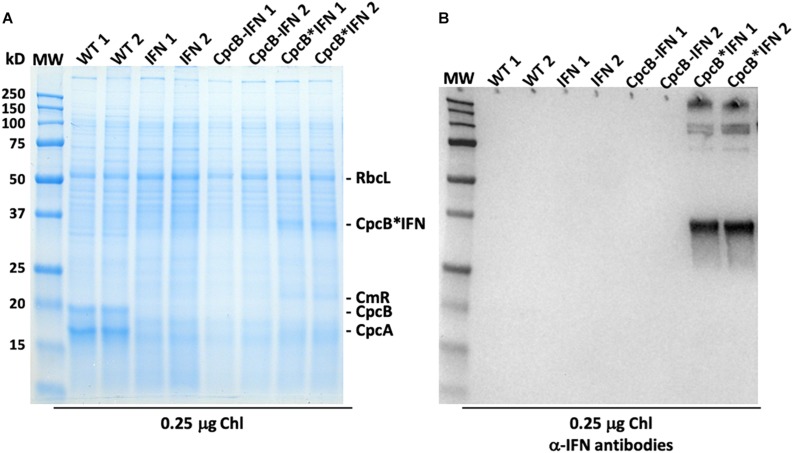
Protein expression analysis of *Synechocystis* wild type and transformants. **(A)** Total cellular protein extracts were resolved by SDS-PAGE and visualized by Coomassie-stain. Two independent replicates of total protein extracts from wild type (WT), and IFN, CpcB-IFN, and CpcB^∗^IFN transformant cells were loaded onto the SDS-PAGE. Individual native and heterologous proteins of interest are indicated to the right of the gel. Sample loading corresponds to 0.25 μg of chlorophyll. Note the clear presence of a heterologous protein migrating to ∼36 kD in the CpcB^∗^IFN fusion extracts. **(B)** Total protein extracts of **(A)** were subjected to Western-blot analysis with loading of the lanes as per Figure **(A)**. Specific polyclonal antibodies against the human IFN protein were used to probe target proteins. Sample loading corresponds to 0.25 μg of chlorophyll. Note the specific antibody cross-reaction with proteins migrating to ∼36 and ∼108 kD in the cpcB^∗^IFN fusion and the absence of a cross reaction with any protein from the IFN and cpcB-IFN transformant cells. The latter do not seem to make/accumulate IFN.

Protein analysis of total cell extracts from WT and transformant *Synechocystis* was implemented through SDS-PAGE followed by Coomassie blue staining and Western blot analysis (Figure 4). Two replicate samples of WT protein extracts showed the presence of CpcB β-subunit and CpcA α-subunit of phycocyanin as the dominant protein bands, migrating to ∼19 and ∼17 kD, respectively. Another dominant band in the SDS-PAGE profile was the large subunit of Rubisco (RbcL), migrating to about ∼56 kD (Figure 4A). The latter was used as a normalization factor in protein quantification and as a loading control of the gels.

CpcB and CpcA subunits were not evident in the protein extracts of the transformants because of inability of these transformants to assemble the phycobilisome-peripheral phycocyanin rods. The *IFN* and *cpcB-IFN* transformants failed to show accumulation of recombinant IFN protein in the expected ∼19 kD region, both in the SDS-PAGE and the associated Western blot (Figure 4B, IFN and CpcB-IFN), suggesting either very-low levels or absence of the recombinant IFN protein from these samples. There results show that the powerful *cpc* promoter was not sufficient to support a measurable IFN (∼19 kD) protein expression/accumulation in *Synechocystis*. On the contrary, protein extracts from the *cpcB^∗^IFN* fusion transformants showed a clear presence of an abundant protein with electrophoretic mobility to ∼36 kD. This band was attributed to accumulation of the CpcB^∗^IFN fusion protein (Figure 4A, CpcB^∗^IFN). Identification of the ∼36 kD protein was tested by Western blot analysis with specific polyclonal antibodies raised against the human IFN protein (Figure 4B, CpcB^∗^IFN). A strong cross-reaction between the polyclonal antibodies and a protein band migrating to ∼36 kD suggested that this band is the recombinant CpcB^∗^IFN protein. Moreover, cross-reactions were also detected with protein bands at a higher MW, suggesting the formation/presence of complexes (∼108 kD) containing the CpcB^∗^IFN fusion protein. The higher MW band (∼250 kD) likely originates from aggregation of proteins containing the fusion CpcB^∗^IFN construct, evidenced by their cross reaction with specific anti-IFN antibodies.

**FIGURE 5:**
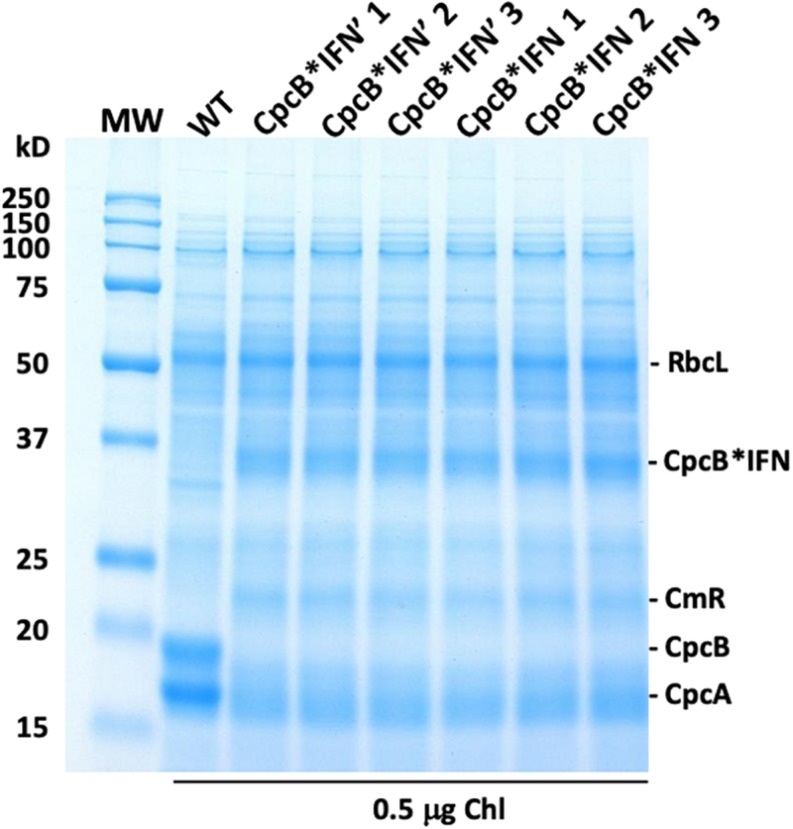
Protein expression analysis of *Synechocystis* wild type (WT) and transformants harboring the *cpcB^∗^IFN* fusion construct. Total cellular protein extracts were resolved by SDS-PAGE and visualized by Coomassie-stain. Two different versions of the IFN gene were used: the human native IFN’ and the *Synechocystis* codon-optimized IFN gene. Note the presence of heterologous proteins migrating to ∼36 kD (CpcB^∗^IFN) and ∼23 kD (CmR) in the transformants but not in the wild type. Also note the presence of the ∼19 kD CpcB β-subunit and the ∼17 kD CpcA α-subunit of phycocyanin in the wild type but not in the transformants. Sample loading corresponds to 0.5 μg of chlorophyll. Quantification of the CpcB^∗^IFN protein accumulation relative to that of the Rubisco large subunit (RbcL) is given in the results of Table 1.

To evaluate the effect of DNA codon-use optimization on the IFN protein expression level, we designed CpcB^∗^IFN fusion DNA constructs using the *Synechocystis* codon optimized IFN as well as the native unoptimized human DNA sequence, termed IFN’ (IFN prime), for comparative expression measurements in *Synechocystis*. The latter construct harbored the same elements of the CpcB^∗^IFN fusion, with the exception of the *IFN* gene that was replaced by the human native *IFN’* sequence (no codon-use optimization). Wild type (WT), *cpcB^∗^IFN’*, and *cpcB^∗^IFN* transformant strains were grown in parallel, total cell proteins were extracted and subjected to SDS-PAGE analysis. Upon Coomassie staining of the SDS-PAGE (Figure 5), the WT protein extract showed as main subunits the 56 kD RbcL, 19 kD CpcB, and 17 kD CpcA. The latter two subunits were missing from the extract of the transformant cells, shown in three independent replicates per transformant in Figure 5. Densitometric analysis of Coomassie stained SDS-PAGE (Figure 5) showed the presence of RbcL to ∼12.5% of total cellular protein. Fusion constructs accumulated to ∼10.2% in the *cpcB^∗^IFN’* and ∼11.8% in *cpcB^∗^IFN* codon-optimized transformant strains. Validation of the Coomassie stained SDS-PAGE protein assignments was obtained through Western blot analysis with specific polyclonal antibodies (not shown). Since protein comprises in cyanobacteria about 50% of the biomass dry cell weight (dcw), it follows that cpcB^∗^IFN’:Biomass = 5.1% (w:dcw) and cpcB^∗^IFN:Biomass = 5.9% (w:dcw).

**FIGURE 6:**
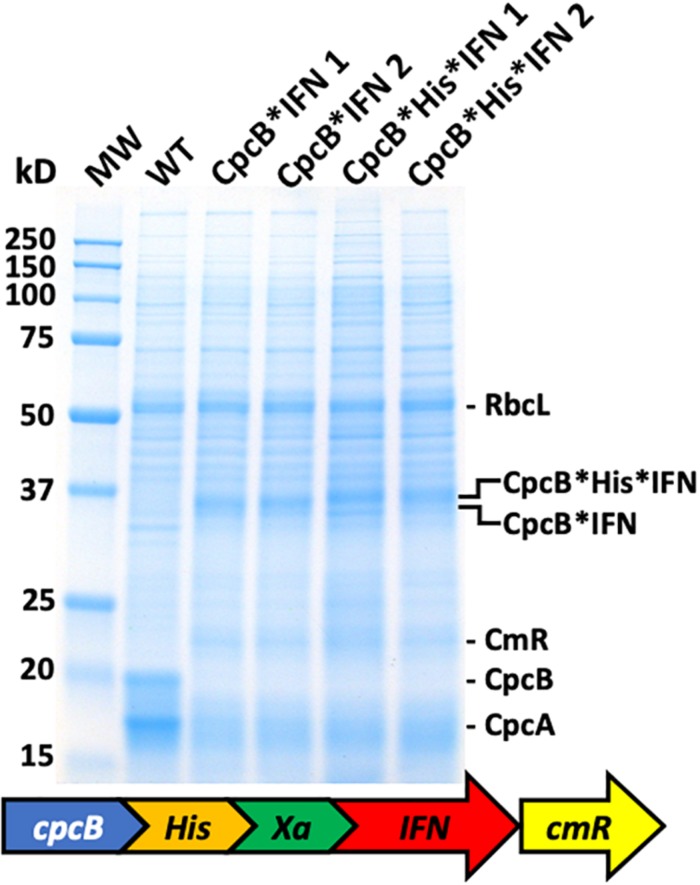
Protein expression analysis of *Synechocystis* wild type (WT) and transformants harboring the *cpcB^∗^His^∗^IFN* fusion construct. Total cellular protein extracts were resolved by SDS-PAGE and visualized by Coomassie-stain. Two different versions of the fusion construct were used comprising the *CpcB^∗^IFN* fusion and the more extensive *cpcB^∗^His^∗^IFN* fusion configuration, followed by the *cmR* resistance cassette. Equivalent amount of the CpcB^∗^IFN and the CpcB^∗^His^∗^IFN fusion proteins were expressed in *Synechocystis*. Individual native and heterologous proteins of interest are indicated to the right of the gel. Sample loading corresponds to 0.25 μg of chlorophyll.

The above results showed that IFN successfully accumulated in *Synechocystis* only when expressed in a fusion construct configuration with the native highly-expressed CpcB subunit of phycocyanin, regardless of whether the *IFN* gene was codon-optimized or not. Aiming to isolate the recombinant fusion protein, we designed a new DNA construct referred to as the *cpcB^∗^His^∗^IFN*, based on the previous CpcB^∗^IFN construct (Figure 6). A DNA fragment encoding the domain of six histidines and the Factor Xa cleavage-site was inserted between the *cpcB* and the *IFN* genes in the fusion construct. Addition of the His-tag between the CpcB and Factor Xa was designed to enable a recovery of the recombinant protein through column purification, whereas the cleavage Xa site would function in the excision of the CpcB^∗^His moiety of the construct, thereby releasing the native form of the target IFN protein (Kavoosi et al., 2007). Protein analysis was then conducted on the homoplasmic transformant lines. Coomassie staining of the SDS-PAGE profile (Figure 6) showed the abundant RbcL, CpcB and CpcA subunits in the wild type extracts (Figure 6, WT). The *cpcB^∗^IFN* transformants lacked the CpcB and CpcA proteins but accumulated the CpcB^∗^IFN as a ∼36 kD protein (Figure 6, CpcB^∗^IFN). The *cpcB^∗^His^∗^IFN* transformants also lacked the CpcB and CpcA proteins and accumulated an abundant protein band with a slightly higher apparent molecular mass than that of the CpcB^∗^IFN (Figure 6, CpcB^∗^His^∗^IFN). This band was attributed to the CpcB^∗^His^∗^IFN protein. The fact that CpcB^∗^His^∗^IFN protein band showed a similar abundance as that of the CpcB^∗^IFN construct suggested that the His-tag addition to the fusion construct did not adversely affect the expression level of this recombinant protein.

### Batch-Based Purification of the CpcB^∗^His^∗^IFN Recombinant Protein

We initially applied a “batch” purification procedure to the recombinant CpcB^∗^His^∗^IFN protein using a His-Select resin (Sigma) and by following the manufacturer’s instructions. The procedure was conducted in Eppendorf tubes, thereby minimizing the amount of resin and cell extract used. Total cell extracts from WT, *cpcB^∗^IFN*, and *cpcB^∗^His^∗^IFN* fusion construct transgenic cells were employed in a side-by-side comparative resin treatment and purification analysis. Prior to incubation with the resin, cellular extracts were incubated on ice for 20 min in the presence of 1% Triton X-100 to disperse cellular aggregates that appeared to interfere with the precipitation of the resin upon centrifugation. Un-solubilized cell debris were pelleted and discarded following a brief centrifugation. The supernatant, containing the cellular protein extracts, was incubated with the resin for 5 min, followed by centrifugation to pellet the resin and any His-tagged proteins bound to it.

**FIGURE 7:**
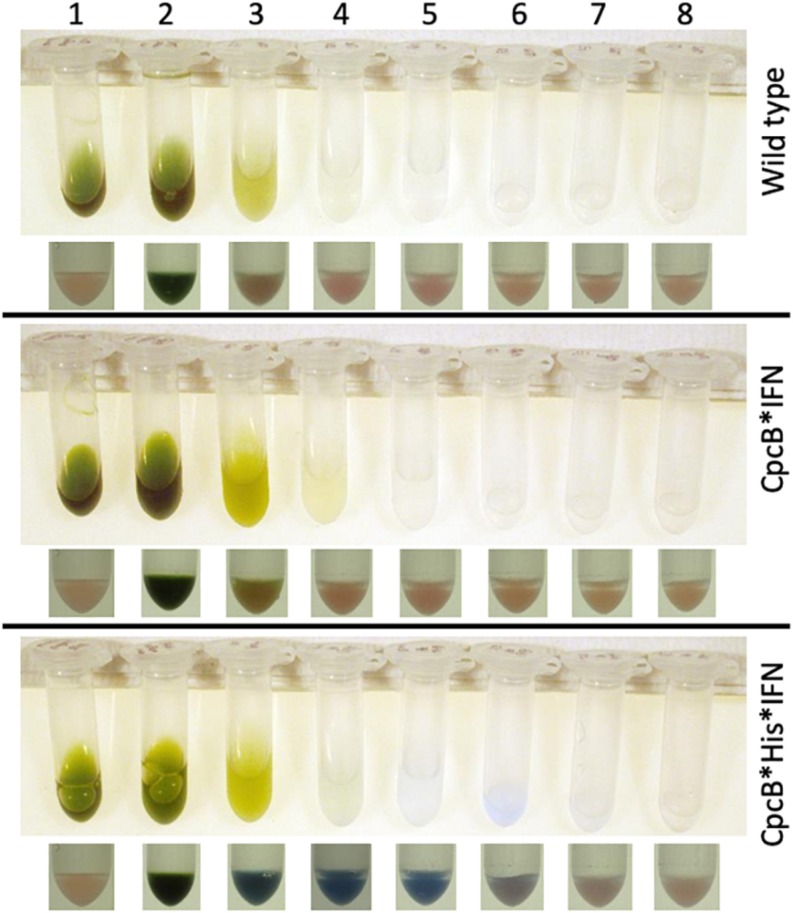
Batch-scale purification of the recombinant CpcB^∗^His^∗^IFN protein through cobalt affinity chromatography. Protein purification was conducted employing a small amount of resin as solid phase. The latter was mixed and incubated with the cell extracts. The resin was pelleted and washed repeatedly with buffers containing imidazole at different concentrations. Lane 1 shows the cell extracts (upper panel) and the resin pellet (lower panel) of the wild type, CpcB^∗^IFN, and CpcB^∗^His^∗^IFN fusion construct cells prior to incubation with the resin. Note the natural pink coloration of the latter. Lane 2 shows the cell extracts (upper panel) and the resin pellet (lower panel) of the wild type, CpcB^∗^IFN, and CpcB^∗^His^∗^IFN fusion construct cells following a 5-min incubation with the resin in the presence of 10 mM imidazole. Note the blue coloration of the resin and the green coloration of the supernatant. Lanes 3–5 show the remaining cell extracts (upper panel) and the resin pellet (lower panel) of the wild type, CpcB^∗^IFN, and CpcB^∗^His^∗^IFN fusion construct cells following a consecutive wash of the resin three times with a buffer containing 10 mM of imidazole. Note the resulting clear supernatant and the pink coloration of the resin after the third wash (lane 5) for the wild type and CpcB^∗^IFN, suggesting absence of His-tagged proteins. Also note the blue coloration of the resin in the CpcB^∗^His^∗^IFN sample, which was retained in this pellet (lanes 3–5) in spite of the repeated wash, suggesting the presence of resin-bound blue-colored His-tagged proteins. Lanes 6–8 show the subsequent extracts (upper panel) and the resin pellet (lower panel) of the wild type, CpcB^∗^IFN, and CpcB^∗^His^∗^IFN fusion construct cells following a wash three times with a buffer containing 250 mM of imidazole, designed to dissociate His-tagged proteins from the resin. Note the bluish supernatant in lanes 6 and 7 and the corresponding loss of the blue color from the resin pellet, suggesting the specific removal of His-tagged proteins from the resin.

Lane 1 in Figure 7 shows the cell extracts (upper panel) and the resin (lower panel) of the wild type, *cpcB^∗^IFN*, and *cpcB^∗^His^∗^IFN* fusion construct transgenic cells prior to mixing cell extracts with the resin. Note the natural pink coloration of the resin.

Lane 2 in Figure 7 shows the cell extracts (upper panel) and the resin pellet (lower panel) of the wild type, *cpcB^∗^IFN*, and *cpcB^∗^His^∗^IFN* cell lines upon mixing cell extracts with the resin, incubating for 5-min, and following a subsequent centrifugation to pellet the resin. Note the blue coloration of the resin pellet and the green coloration of the supernatant. The blue coloration of the cpcB^∗^fusion constructs, including the *cpcB^∗^IFN* and *cpcB^∗^His^∗^IFN*, is attributed to the covalent binding of blue-colored phycobilin pigments to the CpcB protein (discussed in more detail in the results below).

Lanes 3–5 in Figure 7 show the remaining cell extracts (upper panels) and the resin pellet (lower panels) of the wild type, *cpcB^∗^IFN*, and *cpcB^∗^His^∗^IFN* cell lines following a consecutive repeated wash of the resin with a buffer containing 10 mM imidazole to remove non-target proteins. Note the clear supernatant and the pink coloration of the resin after the third wash (lane 5) for the wild type and *cpcB^∗^IFN* transformants, suggesting absence of His-tagged proteins. Also note the blue coloration of the resin in the *cpcB^∗^His^∗^IFN* sample, which was retained in this pellet (lanes 3–5) in spite of the repeated 10 mM imidazole wash, suggesting the presence and binding to the resin of blue-colored His-tagged proteins.

Lanes 6–8 in Figure 7 show the subsequent extracts (upper panel) and the resin pellet (lower panel) of the wild type, *cpcB^∗^IFN*, and *cpcB^∗^His^∗^IFN* cell lines following a wash of the resin three times with a buffer containing 250 mM of imidazole, designed to dissociate His-tagged proteins from the resin. Note the bluish supernatant in lanes 6 and 7 of the *cpcB^∗^His^∗^IFN* cell lines only and the loss of the blue color from the corresponding resin pellet, suggesting a specific removal of His-tagged proteins from the resin under these conditions.

**FIGURE 8:**
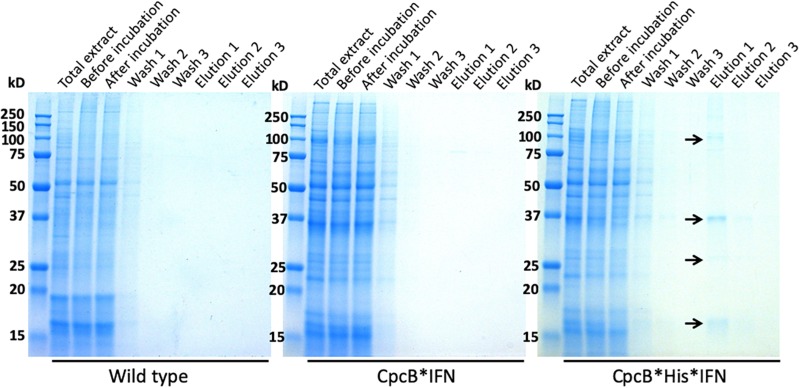
Coomassie-stained SDS-PAGE gel analysis of fractions eluted with different imidazole concentrations. Fractions were obtained upon affinity chromatography purification as shown in Figure 7. Samples were loaded on a per volume basis. Note the ∼108, ∼38, and ∼17 kD proteins eluted from the CpcB^∗^His^∗^IFN extract (marked with arrows). Total cell extract loading corresponds to 0.25 μg of Chl.

Fractions eluted from the resin upon application of 250 mM imidazole were analyzed by SDS-PAGE (Figure 8). Elution fractions from both WT and the *cpcB^∗^IFN* transgenic extracts showed no protein bands in the Coomassie stained gels (Figure 8, left and middle panels), whereas eluent 1 (E1) from the *cpcB^∗^His^∗^IFN* extracts clearly showed the presence of protein bands, with the most abundant migrating to ∼36 kD, attributed to the CpcB^∗^His^∗^IFN fusion protein. Secondary bands migrating to ∼17, ∼27, and ∼108 kD were also noted (Figure 8, right panel). The ∼17 kD protein was attributed to the CpcA α-subunit of phycocyanin. The ∼27 kD protein could be the CpcG1 subunit of the phycobilisome, a phycocyanin rod-core linker polypeptide (Kondo et al., 2005), and the ∼108 kD band is tentatively attributed to a CpcB^∗^His^∗^IFN trimer, as it was shown to contain the CpcB^∗^His^∗^IFN fusion protein (see Figure 4B, also below). These results suggest that CpcA and CpcG1 assemble with the CpcB^∗^His^∗^IFN, potentially as a CpcB^∗^His^∗^IFN-CpcA-CpcG1 complex, in a manner analogous to the α-β phycocyanin heterodimer assembly in the wild type, with the CpcG1 serving as a linker polypeptide, so that the complex binds to the column and elutes together from the resin upon application of 250 mM imidazole.

**FIGURE 9:**
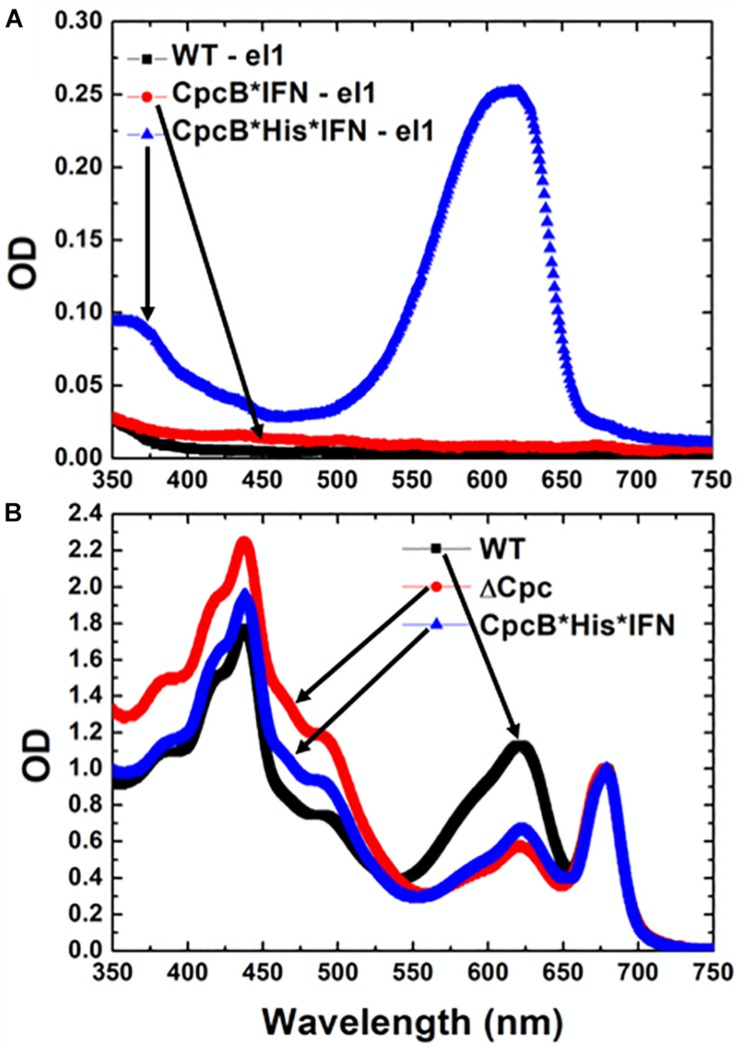
Absorbance spectra of purified *Synechocystis* complexes. **(A)** Absorbance spectra of eluent E1 fractions from wild type, CpcB^∗^IFN, and CpcB^∗^His^∗^IFN samples, as shown in Figure 8. **(B)** Absorbance spectra of cellular protein extracts from wild type, Δcpc deletion mutant (Kirst et al., 2014) and CpcB^∗^His^∗^IFN transformant cells.

The nature of the pigmentation of proteins from elution fraction 1 of the cell extracts was investigated through spectrophotometric analysis (Figure 9A). The spectra of elution fraction 1, referred to as E1, from the WT and CpcB^∗^IFN extracts did not show any absorbance features, consistent with absence of coloration in lanes 6–8 (Figure 7) of these samples. Eluent 1 from the CpcB^∗^His^∗^IFN sample showed a distinct absorbance band with a peak at ∼625 nm and a secondary broad band peaking in the UV-A region of the spectrum. This closely resembled the absorbance spectrum of phycocyanin from *Synechocystis* (Kirst et al., 2014), suggesting the presence of bilin pigment covalently-bound to protein(s) from the CpcB^∗^His^∗^IFN cell extracts. To further investigate this observation, absorbance spectra of total protein extracts from WT and *cpcB^∗^His^∗^IFN* transformant cells were also measured. These were compared with the absorbance spectrum of cells lacking phycocyanin due to a Δcpc operon deletion (Kirst et al., 2014). The spectrum of WT cells showed typical absorbance bands of chlorophyll at 680 nm and phycocyanin at 625 nm (Figure 9B, black line). The extract from the Δcpc transformants showed the specific Chl absorbance peak at 680 nm, whereas the phycocyanin absorbance peak at around 625 nm was missing (Figure 9B, red line). The absorbance spectrum from the *cpcB^∗^His^∗^IFN* transformant cells showed a substantially lower absorbance at about 625 nm due to depletion of phycocyanin, but this decrease was not as extensive as that observed with the Δcpc cells (Figure 9B, blue line). The difference, and apparent low-level absorbance of the *cpcB^∗^His^∗^IFN* cells at 625 nm, suggests that the CpcB protein, albeit in a fusion construct configuration with the IFN, and/or the CpcA protein that apparently accompanies this recombinant protein, manage to covalently bind at least some of the phycobilin pigment that is naturally associated with it, and which is manifested in the blue coloration of the E1 eluent.

### Column-Based Purification of the CpcB^∗^His^∗^IFN Recombinant Proteins

**FIGURE 10:**
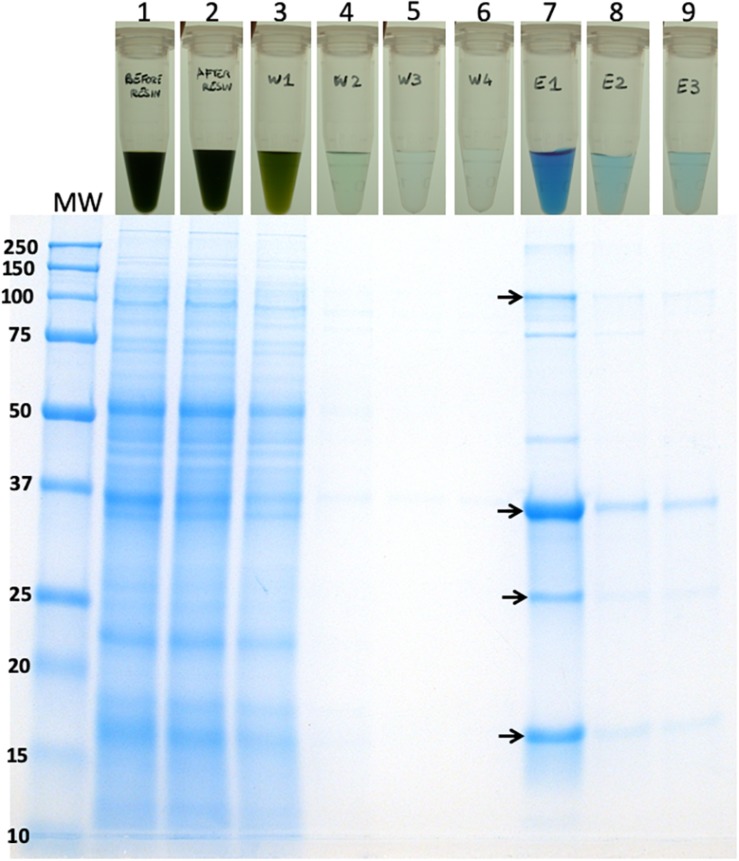
Column-based purification of the CpcB^∗^His^∗^IFN fusion protein through cobalt affinity chromatography. Lane 1, upper panel, shows the CpcB^∗^His^∗^IFN cell extracts in the presence of 5 mM imidazole prior to resin application. Lane 1, lower panel, shows the SDS-PAGE protein profile of these extracts, indicating presence of all *Synechocystis* proteins. Lane 2, upper panel, shows the CpcB^∗^His^∗^IFN cell extracts after incubation with the resin but prior to washing with additional imidazole applications. Lane 2, lower panel, shows the SDS-PAGE protein profile of these extracts, obtained upon a prior removal of the resin from the mix, indicating presence of all *Synechocystis* proteins. Lanes 3–6, upper panel, show the CpcB^∗^His^∗^IFN cell extracts that passed through the resin upon four consecutive washes with 5 mM imidazole and, lower panel, the SDS-PAGE protein profile of these extracts, showing a steep depletion (from lane 3 to lane 6) of total protein. Lanes 7–9, upper panel, show the further removal of resin-bound proteins from the CpcB^∗^His^∗^IFN cell extracts that eluted upon three consecutive washes with 250 mM imidazole and, lower panel, the SDS-PAGE protein profile of these extracts, showing substantial enrichment in mainly four proteins with apparent molecular weights of 108, 36, 27, and 17 kD. The majority of these proteins were eluted with the first application of the 250 mM imidazole solution.

Based on the initial encouraging results obtained with the “batch” purification approach, we proceeded to conduct a “column-based” purification of the His-tagged proteins (Figure 10). This experimental work was conducted as an alternative method in an attempt to elute a greater amount of the CpcB^∗^His^∗^IFN protein. Total protein extract from the *cpcB^∗^His^∗^IFN* transformant cells, mixed with 5 mM imidazole, was loaded onto the resin. Four subsequent washing steps were conducted with 10 mM imidazole to remove non-target proteins from the resin. After these washing steps, elution of the target protein with 250 mM imidazole was undertaken. The pigmentation pattern of the resulting fractions was in accordance with the results obtained with the “batch-based” purification (please see below).

Lane 1 in Figure 10, upper panel, shows the *cpcB^∗^His^∗^IFN* cell extracts that were incubated in the presence of 5 mM imidazole prior to loading on the resin. Lane 1 in Figure 10, lower panel, shows the SDS-PAGE protein profile of these extracts, indicating presence of all expected *Synechocystis* proteins.

Lane 2 in Figure 10, upper panel, shows the *cpcB^∗^His^∗^IFN* cell extracts upon loading and elution from the column but prior to washing with additional imidazole. Lane 2 in Figure 10, lower panel, shows the SDS-PAGE protein profile of these extracts, obtained upon removal of the resin from the mix, again indicating presence of all expected *Synechocystis* proteins.

Lanes 3–6 in Figure 10 (upper panel) show the *cpcB^∗^His^∗^IFN* cell extracts that were eluted from the resin upon four consecutive washes with 10 mM imidazole and (Figure 10, lower panel) the SDS-PAGE protein profile of these extracts, showing removal of the majority of cellular proteins in the first wash (Figure 10, lane 3) and the virtual absence of cell proteins in three additional (lane 4 to lane 6) wash steps with 10 mM imidazole.

**FIGURE 11:**
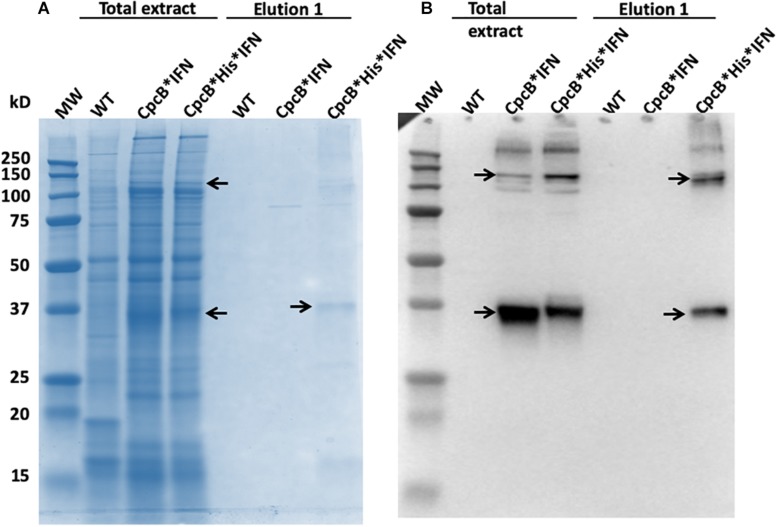
**(A)** SDS-PAGE and Coomassie-staining analysis of *Synechocystis* wild type, CpcB^∗^IFN, and CpcB^∗^His^∗^IFN total cell extract, and of proteins eluted from the resin column upon application of 250 mM imidazole. **(B)** Western blot analysis with specific IFN polyclonal antibodies of the proteins resolved in **(A)**. Note the heterologous ∼36 kD CpcB^∗^His^∗^IFN and the ∼108 kD putative CpcB^∗^His^∗^IFN trimer (marked by arrowheads).

Lanes 7–9 in Figure 10 (upper panel) show the further removal of bound His-tagged proteins from the *cpcB^∗^His^∗^IFN* cell extracts. These eluted from the resin upon three consecutive washes with 250 mM imidazole. Figure 10 (lower panel) is the SDS-PAGE protein profile of these extracts, showing substantial enrichment in mainly four proteins with apparent molecular weights of ∼108, 36, 27, and 17 kD. The majority of these proteins were eluted upon the first application of the 250 mM imidazole (Figure 10, lane 7), as subsequent elution treatments (Figure 10, lanes 8 and 9) produced much lower levels of protein eluent. Western blot analysis with specific anti-IFN antibodies showed strong cross reactions with the 36 and 108 kD protein bands only (Figure 11). The ∼17 kD protein was attributed to the CpcA α-subunit of phycocyanin, as it cross-reacted with CpcA-specific antibodies (not shown, but see also below), whereas the 27 kD protein was attributed to the CpcG1 linker polypeptide (Kondo et al., 2005) that helped to bind the CpcA α-subunit to the CpcB^∗^His^∗^IFN fusion complex, thereby explaining the simultaneous elution of all three proteins from the resin.

### Blue Coloration of the Target Proteins

**FIGURE 12:**
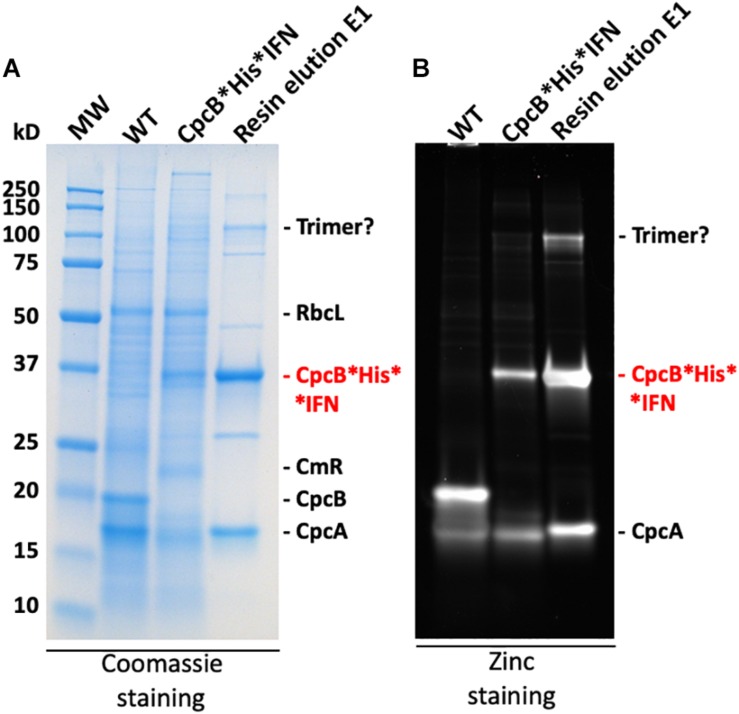
**(A)** SDS-PAGE and Coomassie-stain analysis of *Synechocystis* wild type, CpcB^∗^His^∗^IFN, and resin-eluted proteins. **(B)** SDS-PAGE and Zinc-stain analysis of *Synechocystis* wild type, CpcB^∗^His^∗^IFN, and resin-eluted proteins. Zn-staining is designed to highlight the presence of bilin tetrapyrrole pigments. Individual native and heterologous proteins of interest are indicated to the right of the gels.

The blue coloration of the target proteins (Figures 7, 10) and the absorbance spectral evidence of Figure 9A, suggested the presence of bilin in association with the recombinant CpcB^∗^His^∗^IFN protein. This finding was surprising as *CpcB^∗^fusion* constructs are known to abolish the assembly of the phycocyanin peripheral rods of the phycobilisome (Formighieri and Melis, 2015, 2016; Chaves et al., 2017; Betterle and Melis, 2018, 2019), leading to the assumption of a CpcB inability to bind bilin. This assumption may not be entirely correct. To further test the spectrophotometric suggestion of bilin presence (Figure 9A), SDS-PAGE analysis of protein extracts from wild type, the *cpcB^∗^His^∗^IFN* transformant, and the resin column-based 1^st^ eluent proteins of the latter (Figure 12A) were subjected to “zinc-staining” (please see section “Materials and Methods”). Zinc-staining is designed to specifically label the open tetrapyrroles that are covalently bound to *Synechocystis* proteins. Figure 12B shows the result of the Zn-staining of proteins in a duplicate gel, as the one shown in Figure 12A. In the WT, Zn-staining occurred for proteins migrating to ∼19 and ∼17 kD, attributed to the native CpcB and CpcA phycocyanin subunits. Zn-staining of the total CpcB^∗^His^∗^IFN transformant cell extract occurred for protein bands migrating to ∼36 and ∼17 kD, attributed to the CpcB^∗^His^∗^IFN and the CpcA proteins, respectively. Zn-staining of the first resin eluent (E1) fraction occurred for protein bands migrating to ∼108, ∼36 and ∼17 kD, putatively attributed to a CpcB^∗^His^∗^IFN trimer, the CpcB^∗^His^∗^IFN monomer and the CpcA proteins, respectively. These results corroborate the evidence based on spectrophotometry and Western blot analysis, clearly showing the presence of bilin in association with the CpcB^∗^His^∗^IFN fusion and residual CpcA proteins.

### *nptI^∗^IFN* Fusion Constructs

**FIGURE 13:**
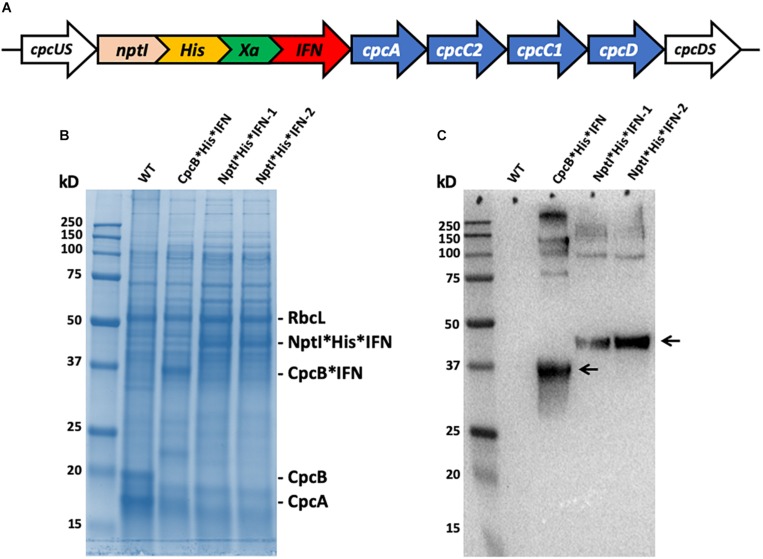
**(A)** Map of the *nptI^∗^IFN* fusion construct in the *cpc* operon locus. Note the presence of the His-tag and the Xa protease cleavage site in-between the two genes in the fusion. **(B)** SDS-PAGE and Coomassie staining of the protein extracts from wild type (WT), the *cpcB^∗^His^∗^IFN*, and two independent lines of the *nptI^∗^His^∗^IFN* transformants. **(C)** Western blot analysis of a duplicate gel as the one shown in **(B)**. Specific anti-IFN polyclonal antibodies were used in this analysis. Note the specific antibody cross reactions with protein bands migrating to ∼36 kD (CpcB^∗^His^∗^IFN) and ∼46 kD (NptI^∗^His^∗^IFN). Also note the antibody cross reactions with protein bands of higher molecular mass.

To further explore the premise of fusion constructs in the expression and accumulation of biopharmaceutical proteins, a new fusion construct was designed for the transformation of wild type (WT) *Synechocystis*, based on the *nptI* gene serving as the leader sequence in a *nptI^∗^IFN* configuration and through homologous DNA recombination in the *cpc* operon (Figure 13A). In such construct, the NptI protein served as the antibiotic selection marker, in addition to being the leader protein sequence in the fusion construct (Betterle and Melis, 2018, 2019). SDS-PAGE analysis of *Synechocystis* protein extracts was conducted (Figure 13B), and *cpcB^∗^His^∗^IFN* transformant showed the expected accumulation of a protein band migrating to about 36 kD (Figure 13B, cpcB^∗^His^∗^IFN). Conversely, two different lines of a transformant expressing the *nptI^∗^His^∗^IFN* construct in the *cpc* operon locus showed the presence of a 46 kD protein attributed to this fusion. Positive identification of these assignments was offered by the Western blot analysis of duplicate gels as the one shown in Figure 13C, further confirming the relative abundance of the fusion constructs expressed in the different *Synechocystis* genomic configurations.

### Relative Antiviral Activity of the Natural IFN and CpcB^∗^His^∗^IFN Fusion Protein

A preliminary effort was undertaken to assess the activity of the cyanobacterial recombinant CpcB^∗^His^∗^IFN protein, as compared with that of commercially-available native interferon (please see section “Materials and Methods”). The results showed that 50 pg/mL of native IFN prevented 50% of cells from becoming infected by the VSV. Conversely, 500 pg/mL of fusion^∗^IFN were needed to prevent 50% of cells from becoming infected by the VSV virus. Part of the difference in sensitivity is probably due to the CpcB leader sequence in the CpcB^∗^His^∗^IFN protein, which may have slowed down the activity of IFN. A consideration in this respect is that the fusion CpcB^∗^His^∗^IFN protein contains about equal molar parts of CpcB^∗^His^∗^Xa (19.4 kD) and IFN (19.2 kD), meaning that the above-mentioned specific activity was underestimated in the case of the fusion protein by a factor of about 2.

## Discussion

Molecular farming, defined as the use of vascular plants for the generation of specialty and commodity products, has been developed and applied, based on the advantage of the process because of minimal requirements of sunlight, carbon dioxide (CO_2_), water, and fertilizer nutrient minerals for growth, simultaneously affording the generation of bio-products and CO_2_ capture and mitigation (Daniell et al., 2001a, b; Ruf et al., 2001; Tregoning et al., 2003; Daniell, 2006; Oey et al., 2009; Matsuo and Atsumi, 2019). Drawbacks of the plant-based system, however, include the slow plant growth and productivity, the low product yields, a lack of guaranteed transgene containment, and the risk of contamination of the human food chain, if edible plant species are used as the host (Wilson and Roberts, 2012). Moreover, transgenic crops used for the production of heterologous proteins would be exposed to agrochemicals and pesticides in the field, while variable culture conditions and the impact of bacterial and fungal infections can lead to fluctuations in yield and product quality (Hellwig et al., 2004; Hidalgo et al., 2017). These plant-based limitations can be alleviated by the cultivation of transgenic cyanobacteria in fully-enclosed photobioreactors, affording better containment, much faster growth, and higher yields.

Recent work from this lab investigated aspects of eukaryotic plant gene transcription, mRNA accumulation, and protein synthesis and stability in cyanobacteria, as these affect the accumulation of heterologous recombinant proteins (Formighieri and Melis, 2016). This is an important issue for the field of synthetic biology as transgenes, and especially eukaryotic transgenes of plant and animal origin, typically are not well-expressed in cyanobacteria (Desplancq et al., 2005, 2008; Formighieri and Melis, 2014, 2015; Jindou et al., 2014). In such efforts, the choice of a strong promoter, such as *cpc*, was necessary but not sufficient to enable high levels of terpene synthase expression in cyanobacteria. Results pointed to the importance of efficient translation for protein accumulation (Formighieri and Melis, 2016). This also appeared to be the case in the present study, pertaining to cyanobacterial expression of interferon.

The promoter of the *cpc* operon controls expression of the abundant phycocyanin subunits and their associated linker polypeptides of the cyanobacterial phycobilisome light-harvesting antenna (Figure 1A). This endogenous strong promoter was employed in an effort to drive heterologous expression of the codon-optimized IFN gene. However, of the three IFN construct configurations (Figures 1B–D), only the fusion construct cpcB^∗^IFN produced substantial amounts of the transgenic IFN protein (Figure 1D). Earlier real time RT-qPCR analysis revealed that such transgene constructs resulted in about equal rates of transcription and showed comparable steady-state levels of eukaryotic transgene mRNA (Formighieri and Melis, 2016). Hence, the rate of transcription does not appear to be the determinant of recombinant protein abundance in this case.

Protein synthesis was later investigated by analyzing the polyribosomes distribution profile associated with the various transcripts (Formighieri and Melis, 2016). A high density of polyribosomes in prokaryotes, such as cyanobacteria, was attributed to a ribosome pileup, when a slower ribosome migration rate on the mRNA causes multiple ribosomes to associate with the same mRNA molecule (Qin and Fredrick, 2013). This was observed to be the case for the Figures 1B,C-type constructs resulting in low transgenic protein accumulation. Conversely, a low density of polyribosomes is attributed to efficient ribosome migration on the mRNA, resulting in efficient translation and high levels of protein accumulation (Qin and Fredrick, 2013). This was observed to be the case for the Figure 1D-type constructs of high transgenic protein accumulation (Formighieri and Melis, 2016).

The significance of codon use optimization for enhancing heterologous protein expression in *Synechocystis* was acknowledged (Lindberg et al., 2010; Vijay et al., 2019). It is noteworthy in this study that codon optimization of the IFN gene (see Materials and Methods) allowed a slight enhancement of the fusion protein expression. Indeed, CpcB^∗^IFN’, with IFN’ prime as the native human gene, accounted for 10.2% ± 0.2 (Figure 5 and Table 1), whereas the CpcB^∗^IFN, with IFN as the *Synechocystis* codon optimized gene accounted for 11.8 ± 0.1% of the total cellular protein. This result corroborated the premise that the main determinant for IFN expression in *Synechocystis* is the protein translation rate, enhanced through the protein fusion technology (Formighieri and Melis, 2016). It may be concluded that promoter strength and codon use optimization may be necessary but not by themselves sufficient to ensure high yield expression.

**TABLE 1:** Quantification of the RbcL and CpcB^∗^IFN fusion proteins as percent of the total *Synechocystis* proteins loaded onto the SDS-PAGE lanes of Figure 5.

**Protein measured**	**CpcB^∗^ IFN’ 1**	**CpcB^∗^ IFN’ 2**	**CpcB^∗^ IFN’ 3**	**CpcB^∗^ IFN 1**	**CpcB^∗^ IFN 2**	**CpcB^∗^ IFN 3**
RbcL	12.1	12.4	13.2	11.9	12.9	12.6
CpcB^∗^IFN	10.4	9.9	10.2	11.8	11.9	11.7

*RbcL levels were measured to account for ∼12.5 ± 0.5%, CpcB^∗^IFN’ accounted for 10.2 ± 0.2%, whereas the CpcB^∗^IFN accounted for 11.8 ± 0.1% of the total cellular proteins.*

It is of interest that elution of the CpcB^∗^His^∗^IFN protein from the corresponding cell lysates showed a bluish coloration, which could serve as a marker for downstream protein processing and purification on an industrial scale. The bluish coloration was shown to be due to the binding of phycocyanobilin to both the CpcB protein in the CpcB^∗^His^∗^IFN recombinant protein and to the small amounts of the phycocyanin α-subunit present. Both of these apparently carry the tetrapyrrole chromophore, as evidenced by the typical phycocyanin absorbance spectra of these extracts (Figure 9A) and by the specific Zn-staining of these proteins (Figure 12). However, it must be noted that, unlike the *in vivo* situation, when about equal amounts of CpcB and CpcA are present (Figure 5, WT), there appears to be no stoichiometry of CpcB^∗^His^∗^IFN and CpcA in the transformants (Figure 5, IFN).

Small amounts of CpcA and of the CpcG1 linker may play a role in stabilizing the CpcB^∗^His^∗^IFN recombinant protein. This contention is supported by the column and resin chromatography, which co-isolated the CpcB^∗^His^∗^IFN fusion along with smaller amounts of the CpcA and CpcG1 linker proteins (Figures 7, 10). This was likely due to the strong protein interactions occurring among phycocyanin subunits (Kondo et al., 2005; Arteni et al., 2009). Such interactions were apparently maintained through the process of cell protein extraction, and through the following mild Triton solubilization and affinity chromatography (see section “Materials and Methods”). Further efforts need be undertaken to chemically/physically disrupt the interactions among the CpcB^∗^His^∗^IFN, CpcA and CpcG1 phycocyanin subunits so as to isolate the CpcB^∗^His^∗^IFN fusion protein in pure form, while maintaining its functionality and bluish coloration.

A question in this work is the possible requirement of IFN glycosylation for function under *in vivo* conditions. According to Adolf et al. (1991), IFN α2 contains a single glycosylation site but recombinant IFNα2 proteins produced in bacteria and cyanobacteria are not glycosylated, as these microorganisms lack the necessary mammalian glycosylation genes and enzymes. However, the glycosylated version of IFN, purified from human leukocytes, shows similar biological activity to the unglycosylated IFN protein (Ghasriani et al., 2013). Furthermore, most of the human IFN-α species are devoid of detectable glycosylation sites (Pestka, 2007), making them suitable for production as biopharmaceuticals at scale in cyanobacteria.

Lastly, of import is the fact that heterologous IFN can also be overexpressed in cyanobacteria with the kanamycin resistance NptI protein in a NptI^∗^His^∗^IFN fusion construct, with the NptI serving as the leader sequence in this configuration (Figure 13). The latter may afford an alternative approach to the purification of the recombinant NptI^∗^His^∗^IFN than that described with the CpcB^∗^His^∗^IFN fusion construct. An advantage in this case would emanate from the fact that NptI, unlike CpcB, would not form a complex with the CpcA and CpcG1 proteins that express along with the CpcB^∗^His^∗^IFN fusion construct (Figures 8, 10). This would simplify the isolation and purification of the recombinant protein. Future experimental work needs to be conducted with the NptI^∗^IFN fusion construct, aiming to investigate alternatives in IFN biosynthesis, improved accumulation of the recombinant protein(s), and downstream processing and isolation of the native IFN from such a *Synechocystis* production process.

## Data Availability Statement

All datasets generated and analyzed in this work are included in the article/Supplementary Material. Included are GenBank accession and protein reference numbers of the genes employed, comprising IFN-α2 (*H. sapiens*), cpcB (*Synechocystis* sp. PCC 6803), cpcA (*Synechocystis* sp. PCC 6803), nptI (*E. coli*), and cmR (*E. coli*) (Supplementary Table S2). Moreover, the codon-optimized nucleotide sequences, as expressed in *Synechocystis* for the purposes of this work, and the nucleotide sequences of the full constructs that were synthesized and employed are also shown in the Supplementary Material, pages 3–8.

## Author Contributions

All authors listed have made a substantial, direct and intellectual contribution to the work, and approved it for publication.

## Conflict of Interest

The authors declare that the research was conducted in the absence of any commercial or financial relationships that could be construed as a potential conflict of interest.

## Footnotes

1 https://www.uniprot.org/uniprot/P01563

2 https://www.idtdna.com/CodonOpt

3 https://www.uniprot.org/uniprot/P01563

## Abbreviations

*cpc*: operon encoding the phycobilisome peripheral phycocyanin antenna
CpcA: α-subunit of phycocyanin
CpcB: β-subunit of phycocyanin
cpcB^∗^IFN: construct containing the cpcB, Xa, and IFN sequences
cpcB^∗^His^∗^IFN: construct containing the cpcB, His-tag, Xa, and IFN sequences in this order
dcw: dry cell weight
IFN: interferon alpha-2
NptI: kanamycin resistance protein
Xa: factor Xa protease cleavage site.
