# Supplementary material for: Cyanobacterial Production of Biopharmaceutical and Biotherapeutic Proteins
Source: Front Plant Sci. 2020 Mar 3;11:237. doi: 10.3389/fpls.2020.00237 (PMC7062967; doi:10.3389/fpls.2020.00237)
Supplement: Supplementary file 1 [file Data_Sheet_1.PDF]

Cyanobacterial production of biopharmaceutical and biotherapeutic proteins

Nico Betterle, Diego Hidalgo Martinez, Anastasios Melis*

Department of Plant and Microbial Biology, University of California, Berkeley, CA 94720-3102, USA

Supplemental and Supporting Information

* Correspondence to: Anastasios Melis, Department of Plant and Microbial Biology, University of California, 111 Koshland Hall, Berkeley, CA 94720-3102, USA; Telephone: 510-642-8166; Fax: 510-642-4995; E-mail: melis@berkeley.edu

Table S1 Sequence of oligonucleotide primers used in the present work.

Oligos name	Oligos DNA sequence
**<*cpc-us for*>**	5'- CCATTAGCAAGGCAAATCAAAGAC -3'
**<*cpcA rev*>**	5'- GGTGGAAACGGCTTCAGTTAAAG -3'

Table S2 Nucleotide GenBank accession and protein reference numbers of the original DNA sequences and proteins used in this work. The referenced GenBank nucleotide sequences are those prior to codon-use optimization for *Synechocystis* expression. The codon-optimized nucleotide sequences, as expressed in *Synechocystis* for the purposes of this work, and the nucleotide sequences of the full constructs that were synthesized and employed here are shown further below, on pages 3-8 in this Supplemental and Supporting Information file.

Gene name	GenBank Accession #	Protein Reference #
IFN-a2 (H. sapiens)	NM_000605.4	NP_000596.2
cpcB **(*Synechocystis* sp PCC 6803)**	NC_020286.1	WP_010871861.1
cpcA **(*Synechocystis* sp PCC 6803)**	NC_000911.1	WP_010871860.1
cpcG1 **(*Synechocystis* sp PCC 6803)**	NC_007775.1	WP_011429130.1
nptI **(*E. coli*)**	NC_012886.1	YP_002995708.1
cmR **(*E. coli*)**	NC_005923.1	YP_025721.1

Construct used for the *IFN-cmR* sequence insertion within the *cpc* operon (*IFN*, codon-optimized human interferon gene for expression in *Synechocystis*), replacing native *cpcB* gene.

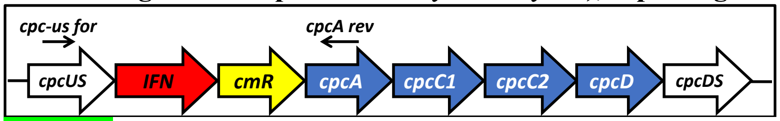

CTCGAG - XhoI DNA restriction site
AGATCT - BglII DNA restriction site
GGATCC - BamHI DNA restriction site

Sequence for 5'- homologous recombination
UPPER CASE ITALICS - *cpc* operon upstream region

UPPER CASE - codon-optimized human interferon gene for expression in *Synechocystis*
lower case - intergenic sequence
UPPER CASE BOLD - *cmR* (chloramphenicol selection cassette)
lower case - Transcription terminator

Sequence for 3'- homologus recombination
UPPER CASE ITALICS - *cpcB-cpcA* intergenic sequence
UPPER CASE BOLD - *cpcA* (partial)

CTCGAG*TAGGCTGTGGTTCCCTAGGCAACAGTCTTCCCTACCCCACTGGAAACTAAAAAAACGAGAAAAGTTCGCAC*
CGAACATCAATTGCATAATTTTAGCCCTAAAACATAAGCTGAACGAAACTGGTTGTCTTCCCTTCCCAATCCAGGACAA
TCTGAGAATCCCCTGCAACATTACTTAACAAAAAAGCAGGAATAAAATTAACAAGATGTAACAGACATAAGTCCCATCA
CCGTTGTATAAAGTTAACTGTGGGATTGCAAAAGCATTCAAGCCTAGGCGCTGAGCTGTTTGAGCATCCCGGTGGCC
CTTGTCGCTGCCTCCGTGTTTCTCCCTGGATTTATTTAGGTAATATCTCTCATAAATCCCCGGGTAGTTAACGAAAGTT
AATGGAGATCAGTAACAATAACTCTAGGGTCATTACTTTGGACTCCCTCAGTTTATCCGGGGGAATTGTGTTTAAGAAA
*ATCCCAACTCATAAAGTCAAGTAGGAGATTAATTCA*ATGTGTGACTTGCCTCAGACGCATTCTTTGGGAAGCCGAC
GCACACTGATGCTGCTCGCCCAAATGCGCCGGATCTCCTTATTCTCCTGTCTCAAGGATCGGCATGACTTCGG
CTTCCCTCAGGAGGAGTTTGGAAATCAGTTCCAAAAGGCCGAAACCATTCCGGTCCTCCATGAAATGATTCAA
CAGATCTTTAACTTATTCAGTACCAAAGACAGCAGTGCGGCCTGGGACGAAACATTACTCGATAAATTCTACA
CGGAATTATACCAACAGTTGAACGACTTAGAAGCCTGTGTAATCCAAGGTGTTGGTGTCACTGAGACTCCATT
AATGAAAGAAGACTCTATTCTGGCCGTCCGCAAGTATTTCCAGCGAATCACACTGTATTTGAAAGAGAAAAA
GTATTCTCCGTGTGCGTGGGAGGTAGTACGGGCTGAAATCATGCGGTCCTTCTCTTTAAGCACAAACCTCCAG
GAATCTCTGCGCTCCAAAGAATGAAGATCTgcggccgcgttgatcggcacgtaagaggttccaactttcaccataatgaaataagatcactaccgggcg
tattttttgagttatcgagattttcaggagctaaggaagctaaa**ATGGAGAAAAAAATCACTGGATATACCACCGTTGATATATCCCAA**
TGGCATCGTAAAGAACATTTTGAGGCATTTCAGTCAGTTGCTCAATGTACCTATAACCAGACCGTTCAG
CTGGATATTACGGCCTTTTTAAAGACCGTAAAGAAAAATAAGCACAAGTTTTATCCGGCCTTTATTCAC
ATTCTTGCCCGCCTGATGAATGCTCATCCGGAATTCCGTATGGCAATGAAAGACGGTGAGCTGGTGAT
ATGGGATAGTGTTCACCCTTGTTACACCGTTTTCCATGAGCAAACTGAAACGTTTTCATCGCTCTGGAG
TGAATACCACGACGATTTCCGGCAGTTTCTACACATATATTCGCAAGATGTGGCGTGTTACGGTGAAAA
CCTGGCCTATTTCCCTAAAGGGTTTATTGAGAATATGTTTTTCGTCTCAGCCAATCCCTGGGTGAGTTT
CACCAGTTTTGATTTAAACGTGGCCAATATGGACAACTTCTTCGCCCCCGTTTTCACCATGGGCAAATA
TTATACGCAAGGCGACAAGGTGCTGATGCCGCTGGCGATTCAGGTTCATCATGCCGTCTGTGATGGCT
TCCATGTCGGCAGAATGCTTAATGAATTACAACAGTACTGCGATGAGTGGCAGGGCGGGGCGTAAtttttta
aggcagttattggtgcccttaaacgcctgg*GGATCCTCTGGTTATTTTAAAAACCAACTTTACTCAGGTTCCATACCCGAGAAAATCCA*
*GCTTAAAGCTGACATATCTAGGAAAATTTTCACATTCTAACGGGAGATACCAGAACA***ATGAAAACCCCTTTAACTG**
AAGCCGTTTCCACCGCTGACTCTCAAGGTCGCTTTCTGAGCAGCACCGAATTGCAAATTGCTTTCGGTC
GTCTACGTCAAGCTAATGCTGGTTTGCAAGCCGCTAAAGCTCTGACCGACAATGCCCAGAGCTTGGTA
AATGGTGCTGCCCAAGCCGTTTATAACAAATTCCCCTACACCACCCAAACCCAAGGCAACAACTTTGCT
GCGGATCAACGGGGTAAAGACAAGTGTGCCCGGGACATCGGCTACTACCTCCGCATCGTTACCTACTG
CTTAGTTGCTGGTGGTACCGGTCCTTTGGATGAGTACTTGATCGCCGGTATTGATGAAATCAACCGCAC
CTTTGACCTCTCCCCCAGCTGGTATGTTCTCGAG

Construct used for the *cpcB-IFN-cmR* sequence insertion within the *cpc* operon (*IFN*, codon-optimized human interferon gene for expression in *Synechocystis*), between *cpcB* and *cpcA* genes.

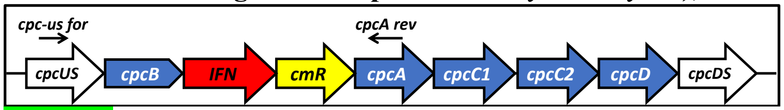

CTCGAG - XhoI DNA restriction site
AGATCT - BglII DNA restriction site
GGATCC - BamHI DNA restriction site

Sequence for 5'- homologus recombination

UPPER CASE - partial *cpcB*
UPPER CASE - *cpcB-cpcA* intergenic sequence

UPPER CASE - codon-optimized human interferon gene for expression in *Synechocystis*
lower case - intergenic sequence
UPPER CASE BOLD - *cmR* (chloramphenicol selection cassette)
lower case - Transcription terminator

Sequence for 3'- homologus recombination

UPPER CASE ITALICS - *cpcB-cpcA* intergenic sequence
UPPER CASE BOLD - *cpcA* (partial)

CTCGAG*CCGCATCACCGGTAATGCTTCCGCTATCGTTTCCAACGCTGCTCGTGCTTTGTTCGCCGAACAGCCCCAAT*
TAATCCAACCCGGTGGAAACGCCTACACCAGCCGTCGTATGGCTGCTTGTTTGCGTGACATGGAAATCATCCTCCGC
TATGTTACCTACGCAACCTTCACCGGCGACGCTTCCGTTCTAGAAGATCGTTGCTTGAACGGTCTCCGTGAAACCTAC
GTTGCCCTGGGTGTTCCCGGTGCTTCCGTAGCTGCTGGCGTTCAAAAAATGAAAGAAGCTGCCCTGGACATCGTTAA
CGATCCCAATGGCATCACCCGTGGTGATTGCAGTGCTATCGTTGCTGAAATCGCTGGTTACTTCGACCGCGCCGCTG
*CTGCCGTAGCCTAG***TCTGGTTATTTTAAAAACCAACTTTACTCAGGTTCCATACCCGAGAAAATCCAGCTT**
AAAGCTGACATATCTAGGAAAATTTTCACATTCTAACGGGAGATACCAGAACAATGTGTGACTTGCCTCA
GACGCATTCTTTGGGAAGCCGACGCACACTGATGCTGCTCGCCCAAATGCGCCGGATCTCCTTATTCTCCTGT
CTCAAGGATCGGCATGACTTCGGCTTCCCTCAGGAGGAGTTTGGAAATCAGTTCCAAAAGGCCGAAACCATT
CCGGTCCTCCATGAAATGATTCAACAGATCTTTAACTTATTCAGTACCAAAGACAGCAGTGCGGCCTGGGACG
AAACATTACTCGATAAATTCTACACGGAATTATACCAACAGTTGAACGACTTAGAAGCCTGTGTAATCCAAG
GTGTTGGTGTCACTGAGACTCCATTAATGAAAGAAGACTCTATTCTGGCCGTCCGCAAGTATTTCCAGCGAAT
CACACTGTATTTGAAAGAGAAAAAGTATTCTCCGTGTGCGTGGGAGGTAGTACGGGCTGAAATCATGCGGTC
CTTCTCTTTAAGCACAAACCTCCAGGAATCTCTGCGCTCCAAAGAATGAAGATCTgcggccgcgttgatcggcacgtaagagg
ttccaactttcaccataatgaaataagatcactaccgggcgtatttttgagttatcgagattttcaggagctaaggaagctaaa**ATGGAGAAAAAAATCACTGGA**
TATACCACCGTTGATATATCCCAATGGCATCGTAAAGAACATTTTGAGGCATTTCAGTCAGTTGCTCAA
TGTACCTATAACCAGACCGTTCAGCTGGATATTACGGCCTTTTTAAAGACCGTAAAGAAAAATAAGCAC
AAGTTTTATCCGGCCTTTATTCACATTCTTGCCCGCCTGATGAATGCTCATCCGGAATTCCGTATGGCA
ATGAAAGACGGTGAGCTGGTGATATGGGATAGTGTTCACCCTTGTTACACCGTTTTCCATGAGCAAACT
GAAACGTTTTCATCGCTCTGGAGTGAATACCACGACGATTTCCGGCAGTTTCTACACATATATTCGCAA
GATGTGGCGTGTTACGGTGAAAACCTGGCCTATTTCCCTAAAGGGTTTATTGAGAATATGTTTTTCGTC
TCAGCCAATCCCTGGGTGAGTTTCACCAGTTTTGATTTAAACGTGGCCAATATGGACAACTTCTTCGCC
CCCGTTTTCACCATGGGCAAATATTATACGCAAGGCGACAAGGTGCTGATGCCGCTGGCGATTCAGGT
TCATCATGCCGTCTGTGATGGCTTCCATGTCGGCAGAATGCTTAATGAATTACAACAGTACTGCGATGA
GTGGCAGGGCGGGGCGTAAtttttttaaggcagttattggtgcccttaaacgcctgg*GGATCCTCTGGTTATTTTAAAAACCAACTTTAC*
TCAGGTTCCATACCCGAGAAAATCCAGCTTAAAGCTGACATATCTAGGAAAATTTTCACATTCTAACGGGAGATACCAG
*AACA***ATGAAAACCCCTTTAACTGAAGCCGTTTCCACCGCTGACTCTCAAGGTCGCTTTCTGAGCAGCAC**
CGAATTGCAAATTGCTTTCGGTCGTCTACGTCAAGCTAATGCTGGTTTGCAAGCCGCTAAAGCTCTGAC
CGACAATGCCCAGAGCTTGGTAAATGGTGCTGCCCAAGCCGTTTATAACAAATTCCCCTACACCACCCA
AACCCAAGGCAACAACTTTGCTGCGGATCAACGGGGTAAAGACAAGTGTGCCCGGGACATCGGCTACT
ACCTCCGCATCGTTACCTACTGCTTAGTTGCTGGTGGTACCGGTCCTTTGGATGAGTACTTGATCGCCG
GTATTGATGAAATCAACCGCACCTTTGACCTCTCCCCCAGCTGGTATGTTCTCGAG

Construct used for the *cpcB*IFN'-cmR* sequence insertion within the *cpc* operon (*IFN'*, not-optimized human interferon gene).

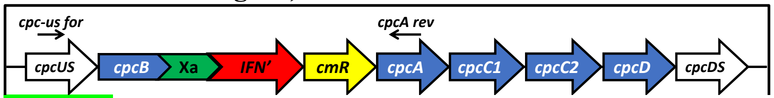

CTCGAG - XhoI DNA restriction site
AGATCT - BglII DNA restriction site
GGATCC - BamHI DNA restriction site

Sequence for 5'- homologus recombination

UPPER CASE ITALICS - *cpcB* (without stop codon)

UPPER CASE - Xa factor cleavage site (IEGR)
UPPER CASE - Native human interferon
lower case - intergenic sequence
UPPER CASE BOLD - *cmR* (chloramphenicol selection cassette)
lower case - Transcription terminator

Sequence for 3'- homologus recombination

UPPER CASE ITALICS - *cpcB-cpcA* intergenic sequence
UPPER CASE BOLD - *cpcA* (partial)

CTCGAG*ATGTTCGACGTATTCACTCGGGTTGTTTCCCAAGCTGATGCTCGCGGCGAGTACCTCTCTGGTTCTCAGTTA*
GATGCTTTGAGCGCTACCGTTGCTGAAGGCAACAAACGGATTGATTCTGTTAACCGCATCACCGGTAATGCTTCCGCT
ATCGTTTCCAACGCTGCTCGTGCTTTGTTCGCCGAACAGCCCCAATTAATCCAACCCGGTGGAAACGCCTACACCAG
CCGTCGTATGGCTGCTTGTTTGCGTGACATGGAAATCATCCTCCGCTATGTTACCTACGCAACCTTCACCGGCGACG
CTTCCGTTCTAGAAGATCGTTGCTTGAACGGTCTCCGTGAAACCTACGTTGCCCTGGGTGTTCCCGGTGCTTCCGTA
GCTGCTGGCGTTCAAAAAATGAAAGAAGCTGCCCTGGACATCGTTAACGATCCCAATGGCATCACCCGTGGTGATTG
*CAGTGCTATCGTTGCTGAAATCGCTGGTTACTTCGACCGCGCCGCTGCTGCCGTAGCC*ATCGAAGGGCGATGTGA
TCTGCCTCAAACCCACAGCCTGGGTAGCAGGAGGACCTTGATGCTCCTGGCACAGATGAGGAGAATCTCTCTT
TTCTCCTGCTTGAAGGACAGACATGACTTTGGATTTCCCCAGGAGGAGTTTGGCAACCAGTTCCAAAAGGCTG
AAACCATCCCTGTCCTCCATGAGATGATCCAGCAGATCTTCAATCTCTTCAGCACAAAGGACTCATCTGCTGC
TTGGGATGAGACCCTCCTAGACAAATTCTACACTGAACTCTACCAGCAGCTGAATGACCTGGAAGCCTGTGTG
ATACAGGGGGTGGGGGTGACAGAGACTCCCCTGATGAAGGAGGACTCCATTCTGGCTGTGAGGAAATACTTC
CAAAGAATCACTCTCTATCTGAAAGAGAAGAAATACAGCCCTTGTGCCTGGGAGGTTGTCAGAGCAGAAATC
ATGAGATCTTTTTCTTTGTCAACAAACTTGCAAGAAAGTTTAAGAAGTAAGGAATGAAGATCTgcggccgcgttgatc
ggcacgtaagaggttccaactttcaccataatgaaataagatcactaccgggcgtattttttgagttatcgagattttcaggagctaaggaagctaaa**ATGGAGAAAAAAA**
TCACTGGATATACCACCGTTGATATATCCCAATGGCATCGTAAAGAACATTTTGAGGCATTTCAGTCAG
TTGCTCAATGTACCTATAACCAGACCGTTCAGCTGGATATTACGGCCTTTTTAAAGACCGTAAAGAAAA
ATAAGCACAAGTTTTATCCGGCCTTTATTCACATTCTTGCCCGCCTGATGAATGCTCATCCGGAATTCC
GTATGGCAATGAAAGACGGTGAGCTGGTGATATGGGATAGTGTTCACCCTTGTTACACCGTTTTCCATG
AGCAAACTGAAACGTTTTCATCGCTCTGGAGTGAATACCACGACGATTTCCGGCAGTTTCTACACATAT
ATTCGCAAGATGTGGCGTGTTACGGTGAAAACCTGGCCTATTTCCCTAAAGGGTTTATTGAGAATATGT
TTTTCGTCTCAGCCAATCCCTGGGTGAGTTTCACCAGTTTTGATTTAAACGTGGCCAATATGGACAACT
TCTTCGCCCCCGTTTTCACCATGGGCAAATATTATACGCAAGGCGACAAGGTGCTGATGCCGCTGGCG
ATTCAGGTTCATCATGCCGTCTGTGATGGCTTCCATGTCGGCAGAATGCTTAATGAATTACAACAGTAC
TGCGATGAGTGGCAGGGCGGGGCGTAAtttttttaaggcagttattggtgcccttaaacgcctgg*GGATCCTCTGGTTATTTTAAAAAC*
CAACTTTACTCAGGTTCCATACCCGAGAAAATCCAGCTTAAAGCTGACATATCTAGGAAAATTTTCACATTCTAACGGG
*AGATACCAGAACA***ATGAAAACCCCTTTAACTGAAGCCGTTTCCACCGCTGACTCTCAAGGTCGCTTTCTGA**
GCAGCACCGAATTGCAAATTGCTTTCGGTCGTCTACGTCAAGCTAATGCTGGTTTGCAAGCCGCTAAAG
CTCTGACCGACAATGCCCAGAGCTTGGTAAATGGTGCTGCCCAAGCCGTTTATAACAAATTCCCCTACA
CCACCCAAACCCAAGGCAACAACTTTGCTGCGGATCAACGGGGTAAAGACAAGTGTGCCCGGGACATC
GGCTACTACCTCCGCATCGTTACCTACTGCTTAGTTGCTGGTGGTACCGGTCCTTTGGATGAGTACTTG
ATCGCCGGTATTGATGAAATCAACCGCACCTTTGACCTCTCCCCCAGCTGGTATGTTCTCGAG

Construct used for the *cpcB*IFN-cmR* sequence insertion within the *cpc* operon (*IFN*, codon-optimized human interferon gene for expression in *Synechocystis*).

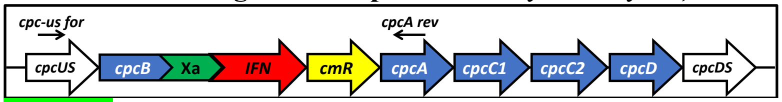

CTCGAG - XhoI DNA restriction site
AGATCT - BglII DNA restriction site
GGATCC - BamHI DNA restriction site

Sequence for 5'- homologus recombination
UPPER CASE ITALICS - *cpcB* (without stop codon)

UPPER CASE - Xa factor cleavage site (IEGR)
UPPER CASE - codon-optimized human interferon gene for expression in *Synechocystis*
lower case - intergenic sequence
UPPER CASE BOLD - *cmR* (chloramphenicol selection cassette)
lower case - Transcription terminator

Sequence for 3'- homologus recombination
UPPER CASE ITALICS - *cpcB-cpcA* intergenic sequence
UPPER CASE BOLD - *cpcA* (partial)

CTCGAG*ATGTTCGACGTATTCACTCGGGTTGTTTCCCAAGCTGATGCTCGCGGCGAGTACCTCTCTGGTTCTCAGTTA*
GATGCTTTGAGCGCTACCGTTGCTGAAGGCAACAAACGGATTGATTCTGTTAACCGCATCACCGGTAATGCTTCCGCT
ATCGTTTCCAACGCTGCTCGTGCTTTGTTCGCCGAACAGCCCCAATTAATCCAACCCGGTGGAAACGCCTACACCAG
CCGTCGTATGGCTGCTTGTTTGCGTGACATGGAAATCATCCTCCGCTATGTTACCTACGCAACCTTCACCGGCGACG
CTTCCGTTCTAGAAGATCGTTGCTTGAACGGTCTCCGTGAAACCTACGTTGCCCTGGGTGTTCCCGGTGCTTCCGTA
GCTGCTGGCGTTCAAAAAATGAAAGAAGCTGCCCTGGACATCGTTAACGATCCCAATGGCATCACCCGTGGTGATTG
*CAGTGCTATCGTTGCTGAAATCGCTGGTTACTTCGACCGCGCCGCTGCTGCCGTAGCC*ATCGAAGGGCGATGTGA
CTTGCCTCAGACGCATTCTTTGGGAAGCCGACGCACACTGATGCTGCTCGCCCAAATGCGCCGGATCTCCTTA
TTCTCCTGTCTCAAGGATCGGCATGACTTCGGCTTCCCTCAGGAGGAGTTTGGAAATCAGTTCCAAAAGGCCG
AAACCATTCCGGTCCTCCATGAAATGATTCAACAGATCTTTAACTTATTCAGTACCAAAGACAGCAGTGCGGC
CTGGGACGAAACATTACTCGATAAATTCTACACGGAATTATACCAACAGTTGAACGACTTAGAAGCCTGTGT
AATCCAAGGTGTTGGTGTCACTGAGACTCCATTAATGAAAGAAGACTCTATTCTGGCCGTCCGCAAGTATTTC
CAGCGAATCACACTGTATTTGAAAGAGAAAAAGTATTCTCCGTGTGCGTGGGAGGTAGTACGGCTGAAATC
ATGCGGTCCTTCTCTTTAAGCACAAACCTCCAGGAATCTCTGCGCTCCAAAGAATGAAGATCTgcggccgcgttgatcg
gcacgtaagaggttccaactttcaccataatgaaataagatcactaccgggcgtatttttttgagttatcgagattttcaggagctaaggaagctaaa**ATGGAGAAAAAAA**
TCACTGGATATACCACCGTTGATATATCCCAATGGCATCGTAAAGAACATTTTGAGGCATTTCAGTCAG
TTGCTCAATGTACCTATAACCAGACCGTTCAGCTGGATATTACGGCCTTTTTAAAGACCGTAAAGAAAA
ATAAGCACAAGTTTTATCCGGCCTTTATTCACATTCTTGCCCGCCTGATGAATGCTCATCCGGAATTCC
GTATGGCAATGAAAGACGGTGAGCTGGTGATATGGGATAGTGTTCACCCTTGTTACACCGTTTTCCATG
AGCAAACTGAAACGTTTTCATCGCTCTGGAGTGAATACCACGACGATTTCCGGCAGTTTCTACACATAT
ATTCGCAAGATGTGGCGTGTTACGGTGAAAACCTGGCCTATTTCCCTAAAGGGTTTATTGAGAATATGT
TTTTCGTCTCAGCCAATCCCTGGGTGAGTTTCACCAGTTTTGATTTAAACGTGGCCAATATGGACAACT
TCTTCGCCCCCGTTTTCACCATGGGCAAATATTATACGCAAGGCGACAAGGTGCTGATGCCGCTGGCG
ATTCAGGTTCATCATGCCGTCTGTGATGGCTTCCATGTCGGCAGAATGCTTAATGAATTACAACAGTAC
TGCGATGAGTGGCAGGGCGGGGCGTAAtttttttaaggcagttattggtgcccttaaacgcctgg*GGATCCTCTGGTTATTTTAAAAAC*
CAACTTTACTCAGGTTCCATACCCGAGAAAATCCAGCTTAAAGCTGACATATCTAGGAAAATTTTCACATTCTAACGGG
*AGATACCAGAACA***ATGAAAACCCCTTTAACTGAAGCCGTTTCCACCGCTGACTCTCAAGGTCGCTTTCTGA**
GCAGCACCGAATTGCAAATTGCTTTCGGTCGTCTACGTCAAGCTAATGCTGGTTTGCAAGCCGCTAAAG
CTCTGACCGACAATGCCCAGAGCTTGGTAAATGGTGCTGCCCAAGCCGTTTATAACAAATTCCCCTACA
CCACCCAAACCCAAGGCAACAACTTTGCTGCGGATCAACGGGGTAAAGACAAGTGTGCCCGGGACATC
GGCTACTACCTCCGCATCGTTACCTACTGCTTAGTTGCTGGTGGTACCGGTCCTTTGGATGAGTACTTG
ATCGCCGGTATTGATGAAATCAACCGCACCTTTGACCTCTCCCCCAGCTGGTATGTTCTCGAG

Construct used for the *cpcB*His*Xa*IFN-cmR* sequence insertion within the *cpc* operon (*IFN*, codon-optimized human interferon gene for expression in *Synechocystis*).

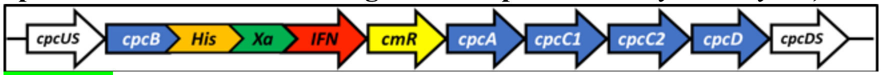

CTCGAG - XhoI DNA restriction site
AGATCT - BglII DNA restriction site
GGATCC - BamHI DNA restriction site

Sequence for 5'- homologus recombination
UPPER CASE ITALICS - *cpcB* (without stop codon)

UPPER CASE - Histag (6x)
UPPER CASE - Xa factor cleavage site (IEGR)
UPPER CASE - codon-optimized human interferon gene for expression in *Synechocystis*
lower case - intergenic sequence
UPPER CASE BOLD - *cmR* (chloramphenicol selection cassette)
lower case - Transcription terminator

Sequence for 3'- homologus recombination
UPPER CASE ITALICS - *cpcB-cpcA* intergenic sequence
UPPER CASE BOLD - *cpcA* (partial)

CTCGAG*ATGTTCGACGTATTCACTCGGGTTGTTTCCCAAGCTGATGCTCGCGGCGAGTACCTCTCTGGTTCTCAGTTA*
GATGCTTTGAGCGCTACCGTTGCTGAAGGCAACAAACGGATTGATTCTGTTAACCGCATCACCGGTAATGCTTCCGCT
ATCGTTTCCAACGCTGCTCGTGCTTTGTTCGCCGAACAGCCCCAATTAATCCAACCCGGTGGAAACGCCTACACCAG
CCGTCGTATGGCTGCTTGTTTGCGTGACATGGAAATCATCCTCCGCTATGTTACCTACGCAACCTTCACCGGCGACG
CTTCCGTTCTAGAAGATCGTTGCTTGAACGGTCTCCGTGAAACCTACGTTGCCCTGGGTGTTCCCGGTGCTTCCGTA
GCTGCTGGCGTTCAAAAAATGAAAGAAGCTGCCCTGGACATCGTTAACGATCCCAATGGCATCACCCGTGGTGATTG
*CAGTGCTATCGTTGCTGAAATCGCTGGTTACTTCGACCGCGCCGCTGCTGCCGTAGCC*CACCATCACCATCACCAT
ATCGAAGGGCGATGTGACTTGCCTCAGACGCATTCTTTGGGAAGCCGACGCACACTGATGCTGCTCGCCCAA
ATGCGCCGGATCTCCTTATTCTCCTGTCTCAAGGATCGGCATGACTTCGGCTTCCCTCAGGAGGAGTTTGGAA
ATCAGTTCCAAAAGGCCGAAACCATTCCGGTCCTCCATGAAATGATTCAACAGATCTTTAACTTATTCAGTAC
CAAAGACAGCAGTGCGGCCTGGGACGAAACATTACTCGATAAATTCTACACGGAATTATACCAACAGTTGAA
CGACTTAGAAGCCTGTGTAATCCAAGGTGTTGGTGTCACTGAGACTCCATTAATGAAAGAAGACTCTATTCTG
GCCGTCCGCAAGTATTTCCAGCGAATCACACTGTATTTGAAAGAGAAAAAGTATTCTCCGTGTGCGTGGGAG
GTAGTACGGGCTGAAATCATGCGGTCCTTCTCTTTAAGCACAAACCTCCAGGAATCTCTGCGCTCCAAAGAAT
GAAGATCTgcggccgcgttgatcggcacgtaagaggttccaactttcaccataatgaaataagatcactaccgggcgtattttttgagttatcgagattttcaggagctaaggaa
gctaaa**ATGGAGAAAAAAATCACTGGATATACCACCGTTGATATATCCCAATGGCATCGTAAAGAACATTT**
TGAGGCATTTCAGTCAGTTGCTCAATGTACCTATAACCAGACCGTTCAGCTGGATATTACGGCCTTTTT
AAAGACCGTAAAGAAAAATAAGCACAAGTTTTATCCGGCCTTTATTCACATTCTTGCCCGCCTGATGAA
TGCTCATCCGGAATTCCGTATGGCAATGAAAGACGGTGAGCTGGTGATATGGGATAGTGTTCACCCTT
GTTACACCGTTTTCCATGAGCAAACTGAAACGTTTTCATCGCTCTGGAGTGAATACCACGACGATTTCC
GGCAGTTTCTACACATATATTCGCAAGATGTGGCGTGTTACGGTGAAAACCTGGCCTATTTCCCTAAAG
GGTTTATTGAGAATATGTTTTTCGTCTCAGCCAATCCCTGGGTGAGTTTCACCAGTTTTGATTTAAACGT
GGCCAATATGGACAACTTCTTCGCCCCCGTTTTCACCATGGGCAAATATTATACGCAAGGCGACAAGGT
GCTGATGCCGCTGGCGATTCAGGTTCATCATGCCGTCTGTGATGGCTTCCATGTCGGCAGAATGCTTAA
TGAATTACAACAGTACTGCGATGAGTGGCAGGGCGGGGCGTAAttttttaaggcagttattggtgcccttaaacgcctgg*GGATC*
CTCTGGTTATTTTAAAAACCAACTTTACTCAGGTTCCATACCCGAGAAAATCCAGCTTAAAGCTGACATATCTAGGAAA
*ATTTTCACATTCTAACGGGAGATACCAGAACA***ATGAAAACCCCTTTAACTGAAGCCGTTTCCACCGCTGACTC**
TCAAGGTCGCTTTCTGAGCAGCACCGAATTGCAAATTGCTTTCGGTCGTCTACGTCAAGCTAATGCTGG
TTTGCAAGCCGCTAAAGCTCTGACCGACAATGCCCAGAGCTTGGTAAATGGTGCTGCCCAAGCCGTTT
ATAACAAATTCCCCTACACCACCCAAACCCAAGGCAACAACTTTGCTGCGGATCAACGGGGTAAAGAC
AAGTGTGCCCGGGACATCGGCTACTACCTCCGCATCGTTACCTACTGCTTAGTTGCTGGTGGTACCGGT
CCTTTGGATGAGTACTTGATCGCCGGTATTGATGAAATCAACCGCACCTTTGACCTCTCCCCCAGCTGG
TATGTTCTCGAG

Construct used for the *nptI*His*Xa*IFN* sequence insertion within the *cpc* operon (*IFN*, codon-optimized human interferon gene for expression in *Synechocystis*).

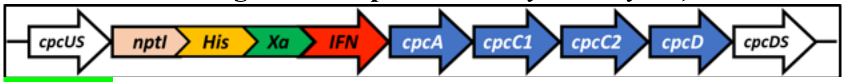

CTCGAG - XhoI DNA restriction site
GGATCC - BamHI DNA restriction site

Sequence for 5'- homologus recombination
UPPER CASE ITALICS - *cpc* operon upstream region

UPPER CASE - *nptI* (kanamycin selection cassette)
UPPER CASE - Histag (6x)
UPPER CASE - Xa factor cleavage site (IEGR)
UPPER CASE - codon-optimized human interferon gene for expression in *Synechocystis*
lower case - Transcription terminator

Sequence for 3'- homologus recombination
UPPER CASE ITALICS - *cpcB-cpcA* intergenic sequence
UPPER CASE BOLD - *cpcA* (partial)

CTCGAG*GGAAAGTAGGCTGTGGTTCCCTAGGCAACAGTCTTCCCTACCCCACTGGAAACTAAAAAAACGAGAAAAGT*
TCGCACCGAACATCAATTGCATAATTTTAGCCCTAAAACATAAGCTGAACGAAACTGGTTGTCTTCCCTTCCCAATCCA
GGACAATCTGAGAATCCCCTGCAACATTACTTAACAAAAAAGCAGGAATAAAATTAACAAGATGTAACAGACATAAGTC
CCATCACCGTTGTATAAAGTTAACTGTGGGATTGCAAAAGCATTCAAGCCTAGGCGCTGAGCTGTTTGAGCATCCCGG
TGGCCCTTGTCGCTGCCTCCGTGTTTCTCCCTGGATTTATTTAGGTAATATCTCTCATAAATCCCCGGGTAGTTAACGA
AAGTTAATGGAGATCAGTAACAATAACTCTAGGGTCATTACTTTGGACTCCCTCAGTTTATCCGGGGGAATTGTGTTTA
AGAAAATCCCAACTCATAAAGTCAAGTAGGAGATTAATTCAATGAGTCACATCCAGAGAGAAACTAGTTGTTCCCGACC
TCGTTTGAATAGCAATATGGATGCAGATCTGTACGGATATAAATGGGCGCGAGATAACGTAGGCCAATCTGGGGCCA
CTATTTATCGGTTATATGGCAAACCAGATGCTCCCGAACTGTTTCTCAAACATGGCAAAGGGTCTGTGGCCAATGATG
TTACCGATGAAATGGTGCGGTTGAACTGGTTGACAGAATTTATGCCCCTCCCGACCATCAAACATTTTATCAGGACTC
CAGACGATGCATGGCTATTAACTACGGCCATTCCTGGGAAAACTGCCTTTCAGGTGTTGGAAGAATATCCCGATTCTG
GTGAGAATATCGTCGATGCGTTAGCGGTTTTTCTAAGACGTCTACATAGCATTCCCGTTTGCAATTGTCCCTTTAATTC
GGACCGGGTGTTCCGCTTGGCGCAGGCTCAGTCCCGGATGAATAACGGTTTGGTAGATGCCTCGGACTTTGATGAT
GAACGGAACGGCTGGCCCGTTGAACAGGTTTGGAAAGAGATGCATAAGCTGCTGCCCTTCTCCCCCGACAGCGTTG
TTACTCATGGAGATTTTTCTCTCGATAATCTGATTTTCGACGAAGGCAAGCTAATTGGCTGTATCGATGTGGGACGGGT
AGGGATTGCGGACCGGTATCAAGACCTAGCAATTTTGTGGAACTGCCTAGGTGAATTTTCCCCCAGCCTACAAAAACG
*GCTGTTTCAAAAATACGGAATCGATAATCCCGACATGAACAAATTACAATTTCATCTGATGCTAGATGAGTTCTTT*CAC
CATCACCATCACCATATCGAAGGGCGATGTGACTTGCCTCAGACGCATTCTTTGGGAAGCCGACGCACACTGA
TGCTGCTCGCCCAAATGCGCCGGATCTCCTTATTCTCCTGTCTCAAGGATCGGCATGACTTCGGCTTCCCTCAG
GAGGAGTTTGGAAATCAGTTCCAAAAGGCCGAAACCATTCCGGTCCTCCATGAAATGATTCAACAGATCTTTA
ACTTATTCAGTACCAAAGACAGCAGTGCGGCCTGGGACGAAACATTACTCGATAAATTCTACACGGAATTAT
ACCAACAGTTGAACGACTTAGAAGCCTGTGTAATCCAAGGTGTTGGTGTCACTGAGACTCCATTAATGAAAG
AAGACTCTATTCTGGCCGTCCGCAAGTATTTCCAGCGAATCACACTGTATTTGAAAGAGAAAAAGTATTCTCC
GTGTGCGTGGGAGGTAGTACGGGCTGAAATCATGCGGTCCTTCTCTTTAAGCACAAACCTCCAGGAATCTCTG
CGCTCCAAAGAATGAtttttttaaggcagttattggtgcccttaaacgcctgg*GGATCCTCTGGTTATTTTAAAAACCAACTTTACTCAGGT*
*TCCATACCCGAGAAAATCCAGCTTAAAGCTGACATATCTAGGAAAATTTTCACATTCTAACGGGAGATACCAGAACA***AT**
GAAAACCCCTTTAACTGAAGCCGTTTCCACCGCTGACTCTCAAGGTCGCTTTCTGAGCAGCACCGAATT
GCAAATTGCTTTCGGTCGTCTACGTCAAGCTAATGCTGGTTTGCAAGCCGCTAAAGCTCTGACCGACAA
TGCCCAGAGCTTGGTAAATGGTGCTGCCCAAGCCGTTTATAACAAATTCCCCTACACCACCCAAACCCA
AGGCAACAACTTTGCTGCGGATCAACGGGGTAAAGACAAGTGTGCCCGGGACATCGGCTACTACCTCC
GCATCGTTACCTACTGCTTAGTTGCTGGTGGTACCGGTCCTTTGGATGAGTACTTGATCGCCGGTATTG
ATGAAATCAACCGCACCTTTGACCTCTCCCCCAGCTGGTATGTTCTCGAG
